# aFold – using polynomial uncertainty modelling for differential gene expression estimation from RNA sequencing data

**DOI:** 10.1186/s12864-019-5686-1

**Published:** 2019-05-10

**Authors:** Wentao Yang, Philip Rosenstiel, Hinrich Schulenburg

**Affiliations:** 1 Evolutionary Ecology and Genetics, Zoological Institute, CAU Kiel, Am Botanischen Garten 9, 24118 Kiel, Germany; 2 Institute for Clinical Molecular Biology, CAU Kiel, Am Botanischen Garten 11, 24118 Kiel, Germany; 3 Max Planck Institute for Evolutionary Biology, Ausgust-Thienemann-Str. 2, 24306 Ploen, Kiel, Germany

**Keywords:** Differential expression analysis, RNASeq, Transcriptomics, Normalization, ABSSeq, DESeq2, Voom, edgeR, baySeq

## Abstract

**Background:**

Data normalization and identification of significant differential expression represent crucial steps in RNA-Seq analysis. Many available tools rely on assumptions that are often not met by real data, including the common assumption of symmetrical distribution of up- and down-regulated genes, the presence of only few differentially expressed genes and/or few outliers. Moreover, the cut-off for selecting significantly differentially expressed genes for further downstream analysis often depend on arbitrary choices.

**Results:**

We here introduce a new tool for estimating differential expression in noisy real-life data. It employs a novel normalization procedure (qtotal), which takes account of the overall distribution of read counts for data standardization enhancing reliable identification of differential gene expression, especially in case of asymmetrical distributions of up- and downregulated genes. The tool then introduces a polynomial algorithm (aFold) to model the uncertainty of read counts across treatments and genes. We extensively benchmark aFold on a variety of simulated and validated real-life data sets (e.g. ABRF, SEQC and MAQC-II) and show a higher ability to correctly identify differentially expressed genes under most tested conditions. aFold infers fold change values that are comparable across experiments, thereby facilitating data clustering, visualization, and other downstream applications.

**Conclusions:**

We here present a new transcriptomics analysis tool that includes both a data normalization method and a differential expression analysis approach. The new tool is shown to enhance reliable identification of significant differential expression across distinct data distributions. It outcompetes alternative procedures in case of asymmetrical distributions of up- versus down-regulated genes and also the presence of outliers, all common to real data sets.

**Electronic supplementary material:**

The online version of this article (10.1186/s12864-019-5686-1) contains supplementary material, which is available to authorized users.

## Background

RNA Sequencing or RNA-Seq has become a popular approach for the analysis of gene expression variation and uses the enormous recent advances in next generation sequencing technology. In contrast to array-based methods, RNA-Seq permits the quantification of gene expression without detailed prior genome information, such as gene annotations. Thus, it is widely used for both classical model organisms and also non-model taxa [1]. A common aim of such RNA-Seq studies is to understand inducible biological functions, usually through the analysis of differential gene expression (DE), based on comparison of gene expression levels between two different biological states, as defined by exprimental treatments, developmental stages, or different tissues.

Current statistical approaches for DE analysis in RNA-Seq rely on fitting the distribution of read counts with probabilistic models. These methods often detect significant DE via an inferred probability value, usually adjusted for multiple testing through false discovery rate (FDR) estimation. FDR procedures highly depend on mean-variance relationships [2–4]. In this context, systematic problems arise when variance levels for individual genes are unrealistically small (e.g. under-estimation by limited sample size) [3, 5, 6]. Small variance values may also often reflect artifacts due to stochastic effects or methodological procedures, yet may result in highly statistically significant DE [6, 7] but simultaneously high type I error and FDR at extremely small fold-change [8–10]. To reduce the number of potential artifacts, additional cut-offs in fold change are commonly used [8–10] and often explicitly warranted, in order to be able to focus on only larger changes for subsequent functional analysis. Commonly used fold change thresholds are values of at least 1.5 or 2.0 [11–13]. Moreover, as false positives of DE are also frequently found for genes with a high coefficient of variation, usually at low expression levels, another cut-off for a minimum expression value or read count is also widely applied [8–10]. Both strategies are not ideal, because they rely on an arbitrary choice of the applied threshold for either minimum fold-change and/or minimum expression value.

Alternative solutions are based on the idea of merging these cut-offs into a single statistical model or by reducing the effect of high coefficients of variation. For example, TREAT for *t*-test analysis of microarray data partially addresses this problem via testing the significance of DE on a given fold-change threshold [11]. DESeq2 utilizes an empirical Bayesian method to shrink log fold change values toward zero in consideration of read count dispersion [2]. GFOLD generalizes fold changes based on the posterior distribution of log fold change for RNA-Seq data without replicates [6]. However, these methods only provide a partial solution to the problem. The approach in TREAT still requires that the user provides a cut-off value for fold change. The DESeq2 approach identifies significant DE via a Wald-test comparison of the standard error of log fold change estimates with a normal distribution, which might still result in false positives with extremely small fold-changes [8–10]. The GFOLD method can only be used for data without replication.

We have previously developed ABSSeq as an analysis tool for RNA-Seq data, in order to solve some of the above problems [10]. ABSSeq is based on absolute read count difference across treatments. Neverhteless, ABSSeq, like other methods, still requires a combination of cut-offs (fold change and *p*-value) to achieve high reliability in DE inference.

Here, we introduce a novel integrated approach (aFold) of normalization and DE estimation to enhance reliability of RNA-Seq data analysis, which overcomes the problem of low variance levels. aFold includes two elements: a normalization method and then a subsequent gene expression analysis approach. For the former, we developed a new method to improve the normalization of RNA-Seq data, which we term qtotal and which uses the overall read count distribution and thus variance differences among samples for data standardization. This approach is not constrained by the assumption of presence of non-DE genes, which is common to the popular normalization methods. Therefore, qtotal is applicable to a wider range of data sets, including those with substantial DE across conditions. For the latter, we introduce a new method for differential gene expression analysis (i.e., accurate estimation of fold change from RNA-Seq data, the “aFold algorithm” *sensu strictu*), which models the uncertainty of read count data between treatments and experiments using a polynomial function. It thereby allows calculation of fold-change values comparable across conditions and provides a statistical framework for evaluating the significance of DE, thus avoiding the problem of choosing specific cut-off values. Using real and simulated datasets, we demonstrate that the aFold tool is more efficient in DE ranking, DE visualization, and FDR reduction than several of the currently available RNA-Seq analysis approaches, including DESeq2 [2] and Voom [5, 14], edgeR [15], baySeq [16], ABSSeq [10] and ROTS [17], which were previously compared by colleagues of us in similar analyses [2, 14, 18]. The new approaches qtotal and aFold are available as part of the ABSSeq package [10].

## Results and Discussion

First, we introduce the new normalization procedure, qtotal, which we implemented in the aFold package and which aims at standardizing read count variation by accommodating the influence of DE on the total number of read count. The performance of qtotal is compared with other normalization methods such as TMM [19], geometric [3], cqn [20], MedpgQ2, and UQpgQ2 [21]. Thereafter, we illustrate the aFold approach to model fold change and assess its statistical significance with the help of real data sets. Performance of aFold is next compared with that of DESeq2, Voom, edgeR, baySeq, ABSSeq and ROTS, always used under default settings. Two of these methods also consider log fold change for DE inference and report moderated (DESeq2) or raw (Voom) fold changes as output. Method performance is evaluated based on three complementary criteria: 1) correct gene ranking, that is the ability to rank truly DE genes ahead of non-DE genes; 2) minimization of errors, in particular FDR and type I error rate as well as sensitivity-FDR assessment; and 3) visualization of reported fold changes. We use different well-studied real data sets to assess the performance of each method (Table 1). Furthermore, we also use simulated data in method evaluation, for which data structure can be efficiently controlled and which have been widely used to evaluate similar DE analysis methods [7, 10, 16, 22–25].

**Table 1:** Overiew of the used real data sets

Set name	Average library size	#Present Genes	Sample size	#DEs^1^	Used for
ABRF	54,822,037	42,613	18	29,013	DE & Type I error
SEQC	66,503,428	44,931	10	17,038	DE
MAQC-II	1,421,992	11,907	14	8387	DE
Modencodefly	13,709,954	13,244	147	–	Type I error
HapMap-CEU	5,187,226	12,410	41	8	DE
Bottomly	4,904,164	13,932	21	1112	DE
PrimePCR	–	20,801	–	16,603	True DE

(−) indicates that the statistics are not applied. (1) the number of DEs represents average DEs reported by aFold, DESeq2 and Voom

### Qtotal as a new approach for normalization of read count data

Read count analysis of RNA-Seq data requires normalization before DE inference in order to reduce possible biases from variation in sequencing depth, library preparation, sequencing in different lanes, or other random factors [19, 26]. A variety of different normalization procedures have been developed, which adjust individual read count values across replicates and treatments to achieve a standardization of:

1. total number of read count (a procedure termed total) as an indicator of sequencing depth; this procedure is however easily influenced by outliers of read count at high expression level and DE [19];
2. number of read count in the lower quartiles (a procedure termed quartile), which was introduced with the baySeq approach to avoid a possible bias due to outliers [16]; this procedure highly depends on sequencing depth that largely impacts quartile function;
3. geometric mean of all read count (called geometric), which is used by DESeq [3] and DESeq2 [2] to reduce the influence of outliers on approximating the total number of sequence reads; this approach is also sensitive to sequencing depth and DE which might alter the total number of expressed genes as well as the geometric mean of read count from all genes (see also below data analysis);
4. Trimmed mean of M values (called TMM), which is implemented in edgeR and is based on the assumption that the majority of genes with high expression are not DE [19].
5. removing technical variability using conditional quantile normalization (called cqn), which removes systematic biases such as GC-content and gene length [20].
6. per gene normalization after per sample median (called MedpgQ2) or upper-quartile global scaling (called UQpgQ2) [21].

In general, all above listed methods rely on the assumption of presence of no or few DE genes, such as majority of high expressed genes (TMM), genes from 1st to 3rd quartile (quartile) or median (geometry). However, this assumption may not apply in some situations. For example, presence of a large number of DE genes (e.g., samples between certain tissues or developmental stages) will disturb the detection of non-DE genes. Asymmetrical DE in up and down regulation will impact median estimation.

Here we introduce a new normalization procedure, termed qtotal, to address this problem. It is based on the idea that true DE alters the overall read count distribution (either more or less dispersed), which is reflected by a change in the coefficient of variation (CV) of reads count across genes, while variation in sequencing depth does not affect the CV [27]. qtotal quantifies differences in CV between samples and then uses this information to adjust sequence library size, thus explicitly taking into account that there is variation in overall DE between samples (see Methods for details). We used data sets from SEQC, ABRF, and MAQC-II to illustrate the potential problems of different normalization procedures (see Datasets for details). These data sets are based on replicated RNA samples of the human whole body (UHR) and brain (BHR) [28, 29] and show different sequencing depths (ABRF>SEQC>MAQC-II, Table 1). They include validated DE genes, assessed by quantitative real-time PCR (qRT-PCR) [30]. We used these validated results to define true and false positives: From among the identified DE genes, true positives are those with a log2 fold change of more than 0.5 in the qRT-PCR validated results, while false positives are those with a log2 fold change of less than 0.2 in the qRT-PCR results. The three data sets show large differences in the number of DE genes of more than 70% (Table 1). Moreover, the BHR data set has a larger number of down-regulated genes than the UHR data set (60% of DE belongs to down-regulation according to the PrimePCR data set under log2 fold change cut-off of 0.5) [10, 18, 31].

**Fig. 1:**
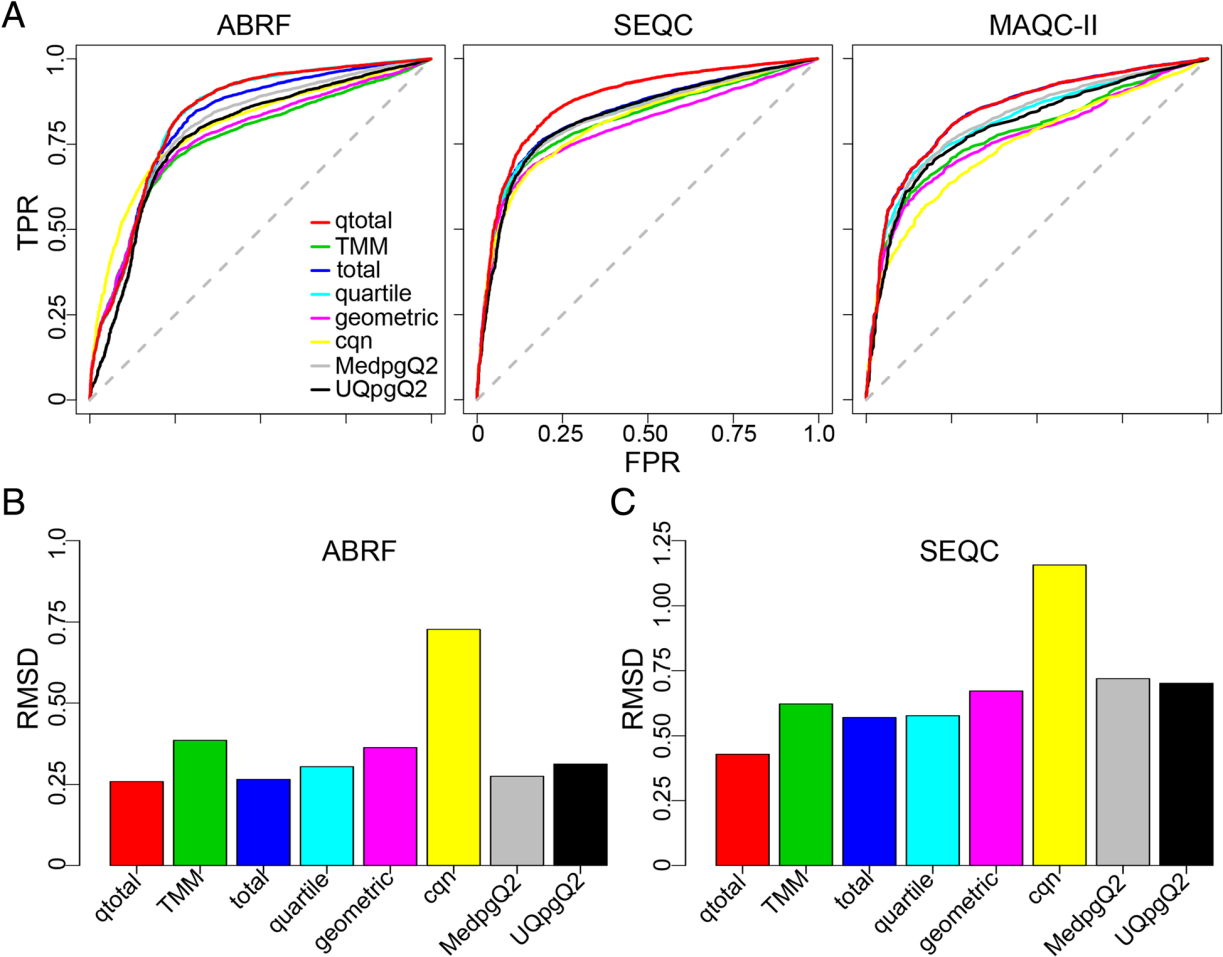
Normalization of RNA-Seq data. (**a**) ROC analysis using the qRT-PCR validated data sets ABRF, SEQC and MAQC-II. ROC analysis for PrimePCR data sets at a qRT-PCR absolute log-ratio (logFC) threshold of 0.5, which results in 9871 true positives. TPR, true positive rate; FPR, false positive rate. A gene is considered to be not differentially regulated if its logFC in the PrimePCR data is less than 0.2, which results in 999 true negatives. Five normalization procedures are analyzed: qtotal, TMM, total, quantile and geometric. ROCs are based on ordinary log fold changes. Rroot mean square deviation (RMSD) correlation of external RNA control consortium (ERCC) for the (**b**) ABRF, and (**c**) SEQC data sets. RMSD is calculated from observed log2 fold change of RNA-Seq data and the expected expected fold change, which results from added RNA markers into samples that mixed into samples UHR and HBR at four ratios: 1/2, 2/3, 1 and 4

The normalization procedures affect the discriminative power of subsequent DE inference. This influence can be assessed with the help of the true and false positive rates (TPR and FPR, respectively) and the area under the Receiver Operating Characteristic (ROC) curve (AUC). The AUCs were inferred with the ROC package in Bioconductor [32], whereby the ROCs were generated based on ordinary fold change under each normalization procedure. We used these three approaches to evaluate the performance of the normalization procedures on the three above listed data sets (Fig. 1a). The performance of the compared methods varies across the three data sets. The discriminative power of the quartile method decreases as the sequence depth decreases (Fig. 1a, from left to right). Normalization with the total of the read count is generally good, indicating that it truly reflects the sequence depth in these three data sets. The TMM and geometric methods perform worse than the other methods except cqn, which might be due to the fact that the majority of genes in the data sets are DE, in apparent contrast to the underlying assumption of the methods. Cqn partially improves the discriminative power on ABRF (beginning of curve), which possibly results from removing biases due to GC-content and gene length. The qtotal method produces significantly larger AUCs on all three data sets (i.e., 0.832, 0.876 and 0.844 for the ABRF, SEQC and MAQC-II data sets, respectively) than the alternative methods (adjusted pvalue < 0.05 via a two sample one-sided z-test [33], see Additional file 1: Table S1 for details). The only two exceptions refer to the quartile approach on the ABRF data set and the total method on the MAQC-II data, which are not signficantly worse than qtotal (Additional file 1: Table S1).

Similar results are also obtained using root mean square deviation (RMSD) analysis of external RNA control consortium (ERCC) data, which compares log2 fold changes with pre-defined fold changes (results from added RNA markers mixed into samples UHR and HBR at four ratios: 1/2, 2/3, 1 and 4) on ABRF (Fig. 1b) and SEQC data set (Fig. 1c). RMSD is used to assess the relationship between expected (e.g., from RT-PCR or spiked-in RNA data) and estimated (e.g., from normalized RNA-Seq data) fold changes [18, 21]. As ERCC data is not available for the MAQC-II data, it was not included in this analysis. The RMSD analysis reveals that qtotal performs best on the two data sets, followed by the total and quartile methods, while cqn yields the worst associations (Fig. 1b, c). When we repeated the RMSD analysis using RNA-Seq and validated PrimePCR data, then, surprisingly, the normalization methods do not differ in their performance (Additional file 1: Figure S1).

Taken together, these results suggest that the qtotal approach is able to normalize RNA-Seq data at least as good as and often with much higher efficacy than alternative procedures. Importantly, its high performance appears to be independent of sequencing depth, as demonstrated by the results on the three real data sets which vary in exactly this parameter (Fig. 1a, Table 1). Therefore, qtotal should improve reliability of subsequent DE detection.

### Benchmarking of aFold with the SEQC and HapMap-CEU data sets

Ordinary fold change indicates the extent of DE for a specific gene, although it is usually not comparable across genes or data sets because of differences in variance. To address this problem, the common idea is to shrink fold changes according to dispersion of read count so that the shrinkage is strong if dispersion for a certain gene is high. DESeq2 employs an empirical Bayes approach to shrink the log fold change according to the mean and dispersion of a gene. The Bayes approach relies on two rounds of fitting a generalized linear model (GLM) to the data: 1) GLM is fitted on read count to obtain maximum-likelihood estimates (MLEs) for the log fold changes and a zero-centered normal distribution of MLEs from all genes; 2) a second GLM is fitted again on the read count data using the zero-centered normal distribution as a prior. Interestingly, the second GLM, which relies on the zero-centered normal distribution of MLEs from all genes, might be influenced by the number of genes with significant DE. If the number of DE genes is high, then the inferred normal distribution shows a flat structure and thus little moderation of fold-change (see below). This could potentially introduce a bias in the obtained fold change values.

aFold estimates fold change through modelling uncertainty of read count data. In particular, the observed read count of RNA-Seq data are characterized by several levels of uncertainty (resulting in observed variance) as a consequence of biological variation, but also due to systematic or non-systematic biases during libarary preparation and sequencing [9, 31]. aFold avoids the implicit assumption of a specific distribution of the read count data (e.g., Poisson or negative binomial, NB [4]). Instead, we explicitly model the uncertainty in the read count data via a polynomial function of the sample mean and standard deviation. aFold takes into account two sources of variance for fold change calculations: 1) the observed variance in gene expression (read count variation across replicates); and 2) the hidden or unknown variance, which is accommodated via fitting the mean-variance relationship (borrowing information from genes). aFold further penalizes high uncertainty of variance estimates, thus ensuring comparability of fold changes across genes and treatments (see Methods section). In contrast to DESeq2, aFold modelling is not influenced by differences between treatments and thus variation in the number of DE genes. Instead, fold change from aFold is a function of the expression level and dispersion of a specific gene.

In addition to estimating fold change itself, aFold also provides an efficient strategy for statistical analysis of DE without the need of defining multiple cut-off values (e.g., fold change in combination with *p*-value and/or minimum expression level). To achieve this, aFold does not directly model read count distributions. Instead, it employs a zero-centered normal distribution on estimated log fold changes and compares them with the global standard deviation (see Methods section). This approach avoids the influence of extremely small variances on significance inference.

**Fig. 2:**
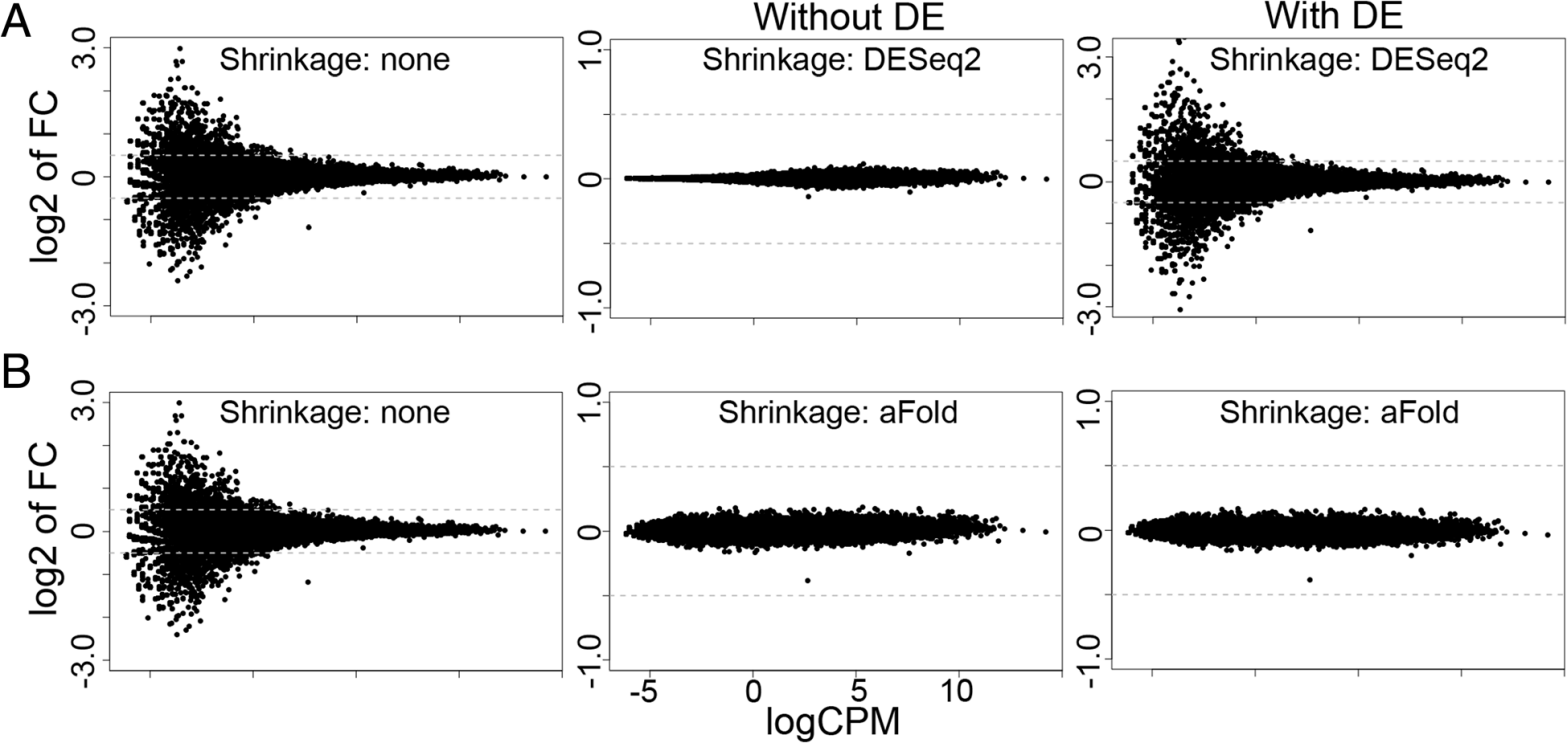
Fold change shrinkage of the aFold and DESeq2 methods. Results are based on the SEQC data set. Fold change is studied for two test comparisons, generated by randomly combining samples from the SEQC data set (four UHR samples). For the first test comparison in (**a**), samples from identical conditions are combined (all UHR), resulting in the absence of true DE (labeled “Without DE”; the left and middle panels). The second test comparison in (**b**) additionally includes pseudo reads count from UHR scaled according to fold change between UHR and BHR, yielding a data set with 40% true DE (labeled “With DE”; right panels). The results are only shown for non-DE genes, in order to enhance comparability between results for data sets with and without DE. This comparison thus allows us to assess the effect of DE genes on normalization efficiency for the non-DE genes. Results for DESeq2 and aFold are based on geometric and qtotal normalization, respectively. They suggest that the presence of a large proportion of DE genes reduces the efficiency of data shrinkage by the approach implemented in DESeq2 but not aFold

The difference in fold-change calculation between DESeq2 and aFold is illustrated in Fig. 2 based on the SEQC data set and calculation of logCPM with the function from the edgeR package [15]. In Fig. 2a and b, four samples in total were randomly selected from this data set (four from UHR) to define two test comparisons. The first of these was set up to contain no true DE by randomly comparing two UHR with two other UHR samples (thus, all data sets coming from identical conditions, labeled “Without DE”; Fig. 2a). This test comparison shows a skewed fold change distribution across different expression levels before application of any fold shrinkage procedure (left panels of Fig. 2a and b). In this case, both DESeq2 and aFold shrink fold change towards zero according to expression level (dispersion) but the shrinkage is stronger in DESeq2 (Fig. 2 a and b, middle panels).

For the second test comparison, we introduced significant DE into the first test comparison. For this, we simulated pseudo reads count from the above used UHR samples, which were scaled according to randomly selected fold changes between UHR and BHR in the SEQC data set, yielding a large number of significant DE, as expected in number and extent for real data sets (because of the differences in tissues). These pseudo reads counts were added to the above used data set, resulting in a data set with about 40% of DE (See source code at https://github.com/wtaoyang/RNASeqComparison for details). For this test comparison, the DESeq2-based shrinkage procedure leads to almost no change in the fold-change distribution and thus no apparent data shrinkage, while that by aFold still results in similar shrinkage as seen for the first test comparison (Fig. 2a and b, right panels). These results, which were obtained from comparable data sets that only varied in the presence or absence of a realistic number of DE genes, suggests that fold change moderation by DESeq2 strongly depends on the number of truly DE genes in the data set, which influences shape of the inferred zero-centered normal distribution. In contrast, moderation of aFold appears to be less affected by DE gene numbers but mainly depends on expression level and dispersion (gene specific and overall dispersion).

**Fig. 3:**
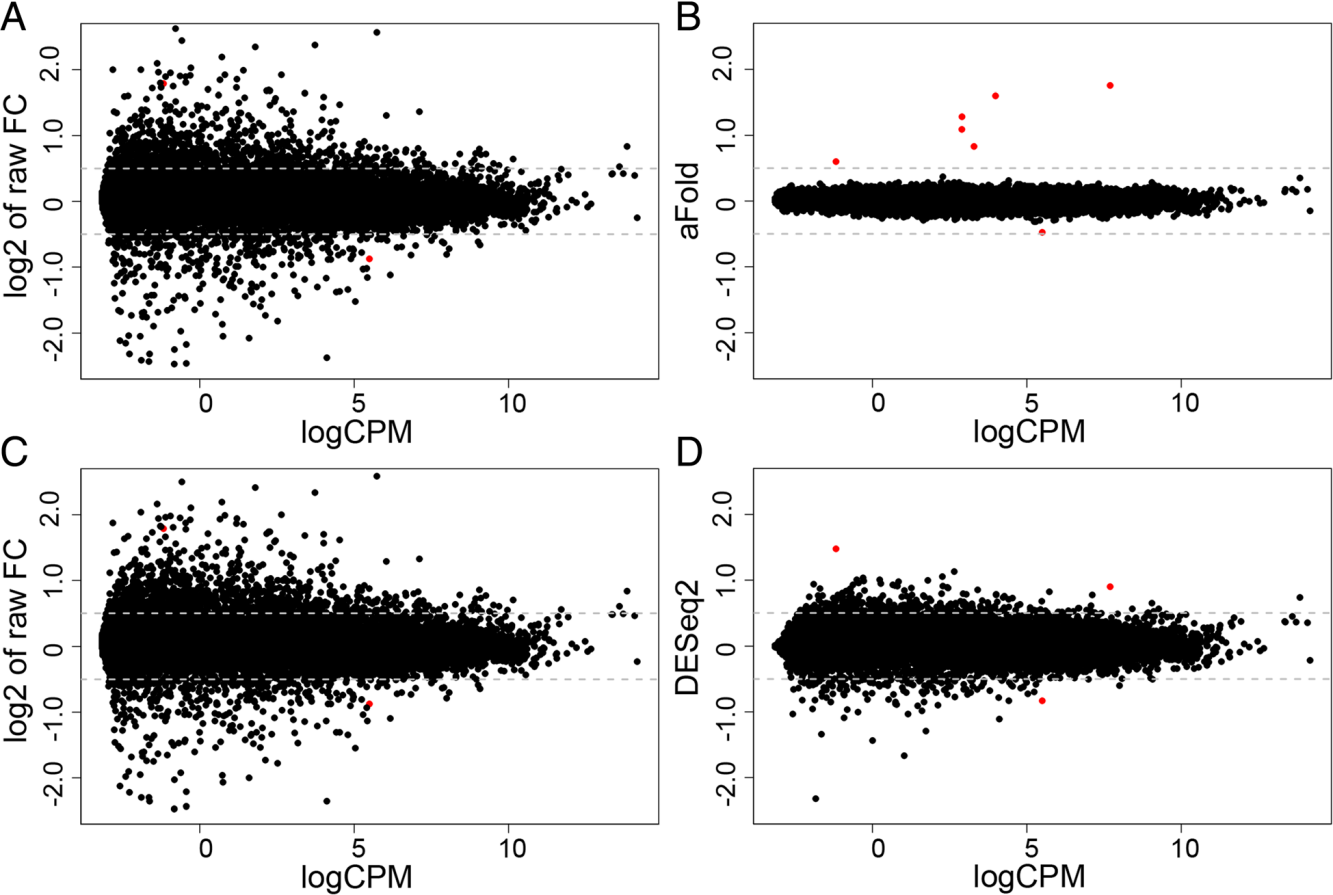
Illustration of the aFold approach with the HapMap-CEU data set. Seven genes on sex chromosomes are marked by red color. (**a**) and (**c**) Raw fold change (without shrinkage) under qtotal and geometric normalization, respectively. Five genes on sex chromosomes are out of y-axis range. (**b**) and (**d**) Fold change values calculated through the aFold (qtotal) and DESeq2 (geometric) approaches, respectively. All seven genes from sex chromosomes show largest fold changes in the aFold result. Four genes on sex chromosomes are out of y-axis range in the DESeq2 result

We next illustrate the aFold approach with the help of the HapMap-CEU data set, which consists of 41 highly dispersed samples from 17 females and 24 males. The HapMap data was split into two group according to gender of patients (male and female). Therefore, the expected or truly DE genes should be sex-related and only includes a total of seven genes on sex chromosomes as highlighted by us previously (identified by all DE methods, see Additional file 1: Table S3 for details) [10]. The results of our analysis are shown in Fig. 3 and the sex-related genes are indicated in red. logCPM was again calculated with the function implemented in the edgeR package [15]. Following [24], a sensitivity analysis is predicted to find an over-representation of inferred DE genes from the sex chromosomes.

The ordinary fold changes between female and male samples exhibit high variability (Fig. 3a) due to high dispersion of the HapMap-CEU data. For the sex-related, truly DE genes, the ordinary fold change values are very large, thus five of the calculated values fall outside of the y-axis range. Such high ordinary fold changes are often produced by genes with low expression level in at least one of the conditions, which often display a high degree of variance. In these cases, the high ordinary fold change does not necessarily reflect the true DE, but represents an artefact resulting from chance effects at very low expression levels. aFold explicitly considers read count variation and expression levels for the calculation of fold change values and thus reports comparable estimates across expression levels. After shrinkage of variance using the aFold approach, fold change values were much smaller and the truly DE genes appear more distinct from the remaining genes (Fig. 3b). In contrast, DESeq2 produces partially shrinked fold changes, with the result that the truly DE genes are not distinct from the non-DE genes (Fig. 3d). These observations may suggest that aFold is able to rank the truly DE before the non-DE genes and produce fold change estimates that directly imply DE.

Statistical assessment of genes with significant DE confirmed the above results (Table 2). Under an adjusted *p*-value cut-off of 0.05, all three considered methods (aFold, DESeq2, Voom) identify seven genes on sex chromosomes with significant DE. If ranked by *p*-value, then all seven sex chromosome genes are within the top 10 DE genes. However, if genes are ranked by fold change, then only aFold is able to find these seven gene within the top 10. These results may suggest that fold changes calculated with aFold are more robust than the conventional fold change calculations (Voom and DESeq2) in ranking truly DE genes. In contrast, consideration of fold-change is not sufficient for identification of truly DE genes by the alternative methods (Voom and DESeq2), but must additionally take into account the associated *p*-vales. Consideration of statistical significance of DE highlights that aFold has similar power than Voom, yet higher specificity in comparison to DESeq2.

**Table 2:** Number of DE genes from sex chromosomes detected by three method in the HapMap-CEU data set at a FDR-adjusted *p*-value of 0.05

Method	Sex^1^/Total^2^	Sex in Top 10 (Rank)	
		p-value	Fold-change
aFold	7/8	7	7
DESeq2	7/12	7	5
Voom	7/7	7	5
edgeR	7/20	7	5
ABSSeq	7/7	7	7
baySeq	7/15	7	5
ROTS-t	7/8	7	5
ROTS-q	7/8	7	5

Number of genes identified by each methods in sex chromosomes (1) and total (2)

### Discrimination of DE versus non-DE genes on qRT-PCR validated real data

We next evaluated the discriminative power of several different methods with the help of three additional data sets. In above Table 2, we showed that aFold is more efficient in ranking true DE before non-DEs. However, the few DE genes of the HapMap-CEU data set might lack resolution to reliably assess method performance. Therefore, we here additionally considered data from the ABRF, SEQC and MAQC-II studies.

The considered ABRF data set consists of RNA-Seq data from the same mRNA sample generated by three different laboratories [9]. This data set includes two conditions (mRNA samples from human whole body and brain), which were sequenced with three replicates at three labs. Therefore, the ARBF data set contains true DE (two conditions) as well as noise (e.g., from library preparation and sequencing), which could be used to assess the accuracy of DE detection approaches, especially their ability to discriminate between signal and noise. Here, we pooled samples for the same condition from three labs into one group (i.e. a comparison of 9:9, nine samples for body and nine for brain). Similarly, the SEQC and MAQC-II data sets contain samples from body and brain but with different sequence depths and number of replicates (See Table 1 and methods for further details). These data sets were used to assess method performance based on TPR and FPR, using ROC curves, resulting AUCs and sensitivity. The AUC has been shown repeatedly to be an informative measure of the overall discriminative power of a method [34–36].

**Fig. 4:**
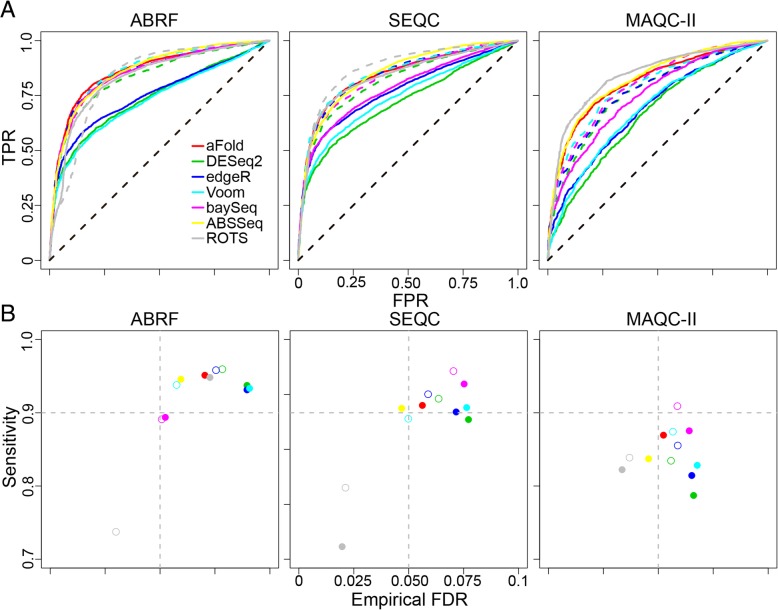
RNA-Seq analysis of the qRT-PCR validated data sets. Analysis is performed on three data sets: ABRF, SEQC and MAQC-II. PrimePCR is used to define true and false positives (TPs, FPs), TPR and FPR are defined as in Fig. 1. (**a**) ROC analysis. Solid lines show the results for the RNA-Seq methods with their integrated normalization procedures. Dashed lines (except diagonal) show the results under qtotal for all methods except for the two methods, aFold and ABSSeq, which use qtotal as default and are thus shown as solid lines. Qtotal improves the performance of most methods. (**b**) Sensitivity analysis. Sensitivity is calculated as the ratio between the number of true DE genes under adjusted *p* value < 0.05 and the total number of true DE genes, inferred from PrimePCR. The empirical false discovery rate (eFDR) is calculated as FPs/(TPs + FPs) under adjusted *p* value < 0.05. qtotal improves either eFDR or sensitivity or both when applied with the tested DE methods. Filled and open circles represent methods with default normalization approach and qtotal, respectively

The results of the analyses are shown in Fig. 4. aFold outperforms the other two methods, irrespective of sequencing depth of data sets (ABRF>SEQC>MAQC-II). aFold reaches the highest AUC values of 0.861, 0.842 and 0.809 on the ABRF, SEQC and MAQC-II, respectively (See Additional file 1: Table S2 for significance). Essentially identical results are obtained, when sequencing depth is artificially varied for one of the data sets, the SEQC data set (Additional file 1: Figure S3), strongly suggesting that the high performance of aFold is independent of sequencing depth. Interestingly, ROC analysis suggests that ranking by fold change is more powerful than *p*-values to detect true DE [8–10]. However, fold changes may fail to indicate DE in highly dispersed data, often genes with low expression (i.e., low FPR, at the beginning of the curve on the ABRF data set). Our model moderates fold changes with information from expression level and dispersion and might be more powerful to detect and rank DE genes than ordinary fold changes and p-values. Notably, aFold performs slightly worse than the ordinary fold change approach on the SEQC and MAQC-II data set in terms of AUCs (0.842 and 0.809 compared with 0.876 and 0.844, respectively) but better on the ABRF data set (0.861 versus 0.836) (Figs. 1 and 4). This might be caused by true DE genes with high dispersion (resulting in strong moderation of fold change), which could be improved by an increased sequencing depth. Strong moderation of fold change might decrease correlation with real fold change (Additional file 1: Figure S4B, RMSD with ERCC), which might require comfirmation from ordinary fold change.

Furthermore, normalization by qtotal further improves the performance of all methods on all three data sets except of the ROTS approach on ABRFAB and MAQC-II and baySeq on ABRFAB (adjusted pvalue < 0.05, see Additional file 1: Table S2 for details). Similar results are obtained when assessing sensitivity versus empirical FDR. qtotal consistently yields higher sensitivity with lower empirical FDR when combined with the various RNA-Seq analysis methods (Fig. 4b). Our additional analysis of SEQC data sets, in which we artifically varied overall read numbers, comfirms that qtotal is able to improve performance of all tested methods, regardless of sequencing depths (Additional file 1: Figure S3). Moreover, qtotal combined with aFold always enhances performance in comparison to aFold used with alternative normalization methods (quartile, geometric and TMM). It also produces slightly better results than originally published combinations of normalization procedures and RNA-Seq analysis methods (i.e., DESeq2 with geometric, Voom and edgeR with TMM and baySeq with quartile) (Additional file 1: Figure S4A).

**Fig. 5:**
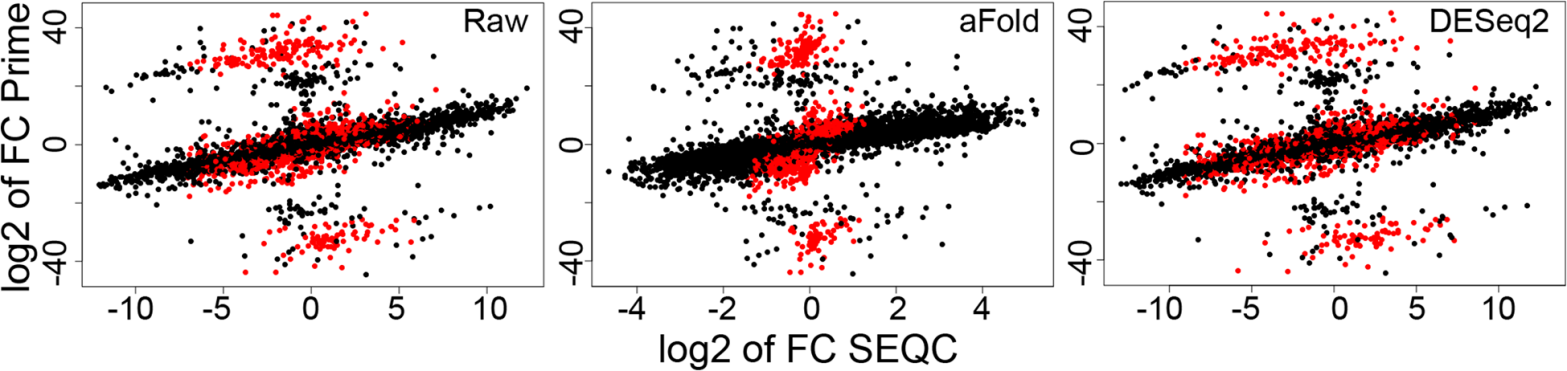
Correlation between fold changes from PrimePCR and SEQC. Scatter plot of log2 fold changes from PrimePCR (y-axis) and SEQC (x-axis). The log2 fold changes for SEQC are generated using qtotal and shown at ordinary (left panel), aFold moderated (middle) and DESeq2 moderated (right) levels. Lowly expressed genes (logCPM < 1) are indicated by red points. The correlation (R-squared) between log2 fold changes from PrimePCR and SEQC increases from 0.18, 0.17 and 0.16 to 0.37, 0.33 and 0.36 by excluding lowerly expressed genes

In addition, we further assessed the relationship between true (PrimePCR) and estimated fold changes (RNA-Seq), as inferred with aFold and DESeq2, using corresponding SEQC data. To avoid any biases, we consistently used qtotal for normalization. Lowly expressed genes (red points in Fig. 5) appear to account for the main differences in fold changes between PrimePCR and SEQC original data. aFold shrinks nearly all fold changes from lowly expressed genes towards zero. In contrast, DESeq2 shrinkage only has a small influence on these genes. Similar results are obtained for comparisons between original and validated ABRF and MAQC-II data sets (Additional file 1: Figure S2B and S2C). These results suggest that aFold produces fold changes that more closely depict true biological variation – at least qualitatively. However, aFold shrinkage also reduces the scale of fold changes from RNA-Seq, which might lead to a decreased correlation with true fold changes in special cases (Additional file 1: Figure S2A, RMSD analysis of ERCC). Nevertheless, fold changes from aFold still preserve the magnitude of DE and even the global correlation with true fold changes (Additional file 1: Figure S2A, RMSD analysis used PrimePCR).

In summary, these results suggest that aFold more efficiently distinguishes between truly DE genes over non-DE genes in real data sets.

### Discrimination of DE versus non-DE genes in simulated data

**Fig. 6:**
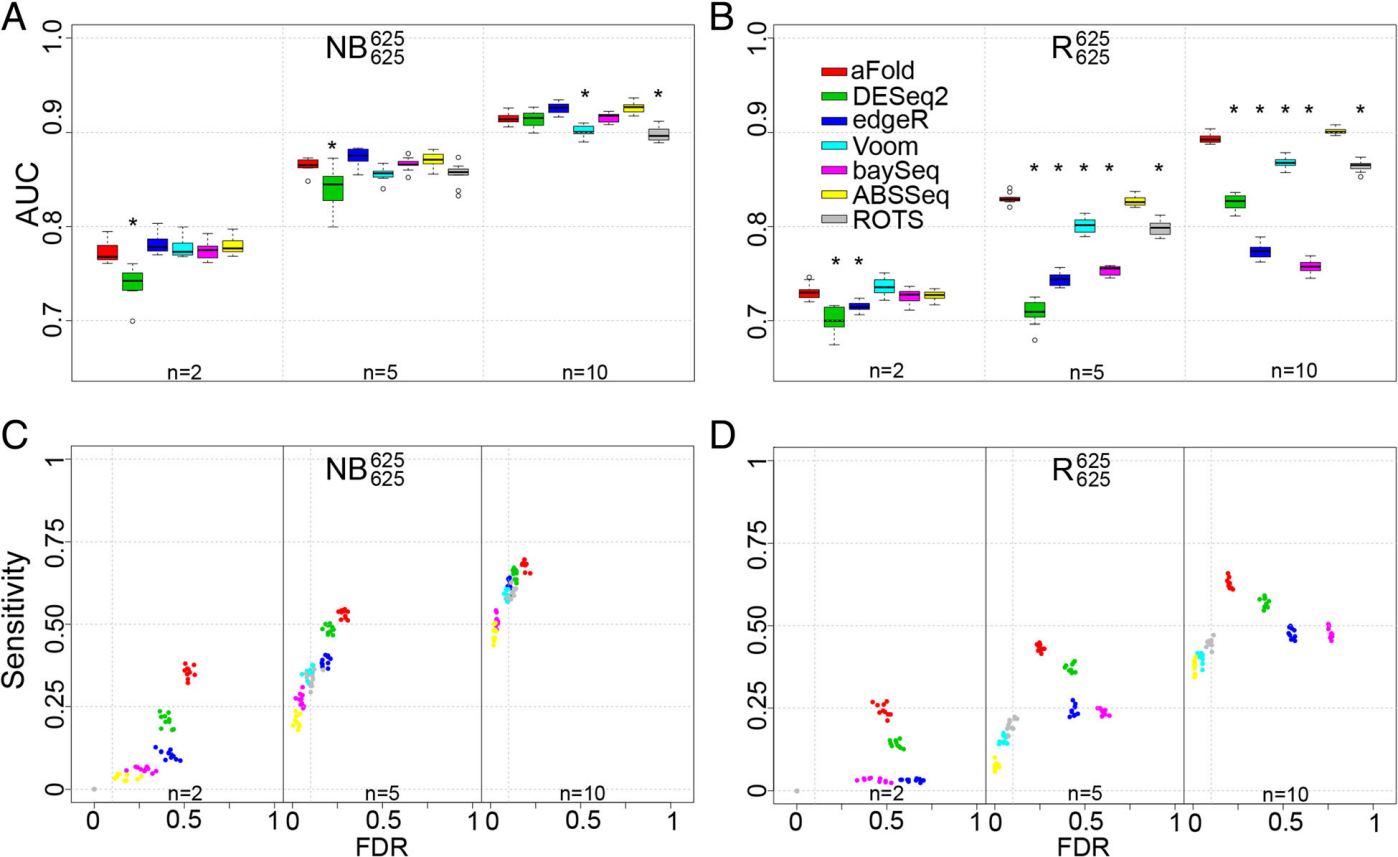
Comparison on simulated data. **a**-**b** Area under the curve (AUC) for aFold and two alternative methods under two simulation settings: (**a**) Negative Binomial (NB) distribution and (**b**) NB distribution with random outliers (R). Each boxplot summarizes the AUCs across 10 independently simulated data sets. Asterisk indicates a statistically significant difference in AUC between aFold and any of the other methods. n indicates the number of considered RNA-Seq replicates, from (2, 5, 10). Under all conditions, aFold is highly effective in correctly identifying differentially expressed genes. **c**-**d** Sensitivity and FDR analysis of the tested methods. The sensitivity is defined as the fraction of genes under adjusted *p*-value < 0.1 among true DEs. The FDR is the fraction of false DEs among genes under adjusted *p*-value < 0.1. ROTS is unable to handle small sample size (*n* = 2) and thus is excluded at *n* = 2

The negative binomial (NB) distribution is most commonly used to increase reliability of DE detection methods as RNA-Seq data shows over-dispersed variance [2, 3, 10, 15]. Here, we evaluated the ability of aFold through ROC analysis on data, which was simulated based on the NB distribution, using mean and variances from Pickrell’s RNA-Seq dataset [37]. For all simulations, we chose 10% of the 12,500 genes to be DE and symmetrically divided them into up- and down-regulated genes (e.g., 625 up- and 625 down-regulated genes, indicated below by super- and subscripts, respectively). We summarize the results using boxplots for two different simulation settings, including data sets with various replicate sample sizes and, in each case, ten independent repetitions (Fig. 6).

When applied on the data that is overdispersed according to the NB distribution (denoted by NB^625^_625_, Fig. 6a), aFold generally yields higher AUCs than alternative methods at large sample size and shows a significant advantage over DESeq2 (*n* = 5) and Voom (*n* = 10) (Tukey’s test, *p* < 0.01). While DESeq2 directly employs a NB model to identify DE, its performance improves as the sample size increases (Fig. 6a). At all three considered sample sizes, aFold produces similar AUC values than alternative methods, suggesting that aFold fits the NB data at least as well as the models used in the other approaches.

Since aFold uses sample variance to calculate fold change and identify DEs, we next tested the influence of outliers that highly impact the sample variance. The outliers were introduced into the NB distributed data by multiplying a randomly generated factor between 5 and 10 with the read count of all genes in all groups obtained through random sampling with a probability of 0.05. The resulting data set (denoted R^625^_625_) still has 625 up- and 625 down-regulated genes, in addition to random outliers. Additional outlier dectection produres were applied for edgeR (GLM_robust) and DESeq2 (Cook’s distance) to analyze R^625^_625_. For these simulated data sets, aFold demonstrates a significant advantage (Tukey’s, *p* < 0.01) over alternative methods at large sample sizes (n = 5 or 10) and even reaches an AUC of 0.9 at n = 10 (Fig. 6b). The only exception refers to the related ABSSeq, which yields similar AUC values than aFold. This result suggests that aFold together with the outlier detection procedure, which we already introduced in ABSSeq, is comparatively mildly affected by outliers. Interestingly, performance of the alternative methods also shows variation. For example, Cook’s distance from DESeq2 requires a high number of replicates to improve its performance in presence of outliers. DE detection of Voom, as implemented in limma, is based on log-transformation, which is more robust against outliers and thus results in higher AUC values than DESeq2. ROTS based on rank statistics is also robust against outliers, which yields similar performance than Voom.

In addition, we also assess the sensitivity and precision (FDR) of all methods with the help of above simulated data sets. As discribed in [2], the sensitivity was calculated as the fraction of genes under adjusted *p*-value < 0.1 among true DEs. The FDR was the fraction of false DEs among genes under adjusted *p*-value < 0.1. The results are shown in Fig. 6c and d for NB^625^_625_ and R^625^_625_, respectively. aFold could always yield higher sensitivity compared to alternative methods. However, aFold also produces slightly higher FDR than other methods on NB^625^_625_. Small sample size (*n* = 2) decreases the power of DE detection for all methods, which leads to either low sensitivity and FDR (e.g. Voom, baySeq and ABSSeq) or high sensitivity but also high FDR (e.g., aFold and DESeq2). Interestingly, in the presence of outliers in the R^625^_625_ data sets, aFold maintains its comparatively high sensitvity and now additionally causes reduced FDR (Fig. 6d), thereby breaking up the usually observed trade-off between sensitivity and FDR.

Similar results are obtained for the simulated data sets with large number of DEs (i.e., 2000 up- and 2000 downregulated genes, NB^2000^_2000_, Additional file 1: Figures. S5A, S6, S7C). Moreover, aFold clearly outperforms alternative methods (except the related ABSSeq method) on data sets simulated with asymmetrical distributions of up- versus down-regulated genes (NB^0^_1250_ and NB^0^_4000_, Additional file 1: Figures. S5 and S7), which is likely a consequence of the efficacy of the qtotal normalization procedure to adjust such biased distributions (Additional file 1: Figure S6). Qtotal is also able to improve the performance of all DE methods on such unbalanced data sets (Additional file 1: Figure S6B-S6F). The only exception refers to Voom, which shows large variation upon qtotal standardization and might be due to gene specific normalization of Voom [18] .

Overall, aFold is at least as good as alternative methods in discriminating between DE and non-DE genes in the presence of outliers, irrespective of the here considered data sample sizes. In combination with qtotal normalization, aFold clearly outperforms alternative methods in case of asymmetrical distributions of up- and down-regulated genes.

### Control of false discovery rate and type I error rate

**Fig. 7:**
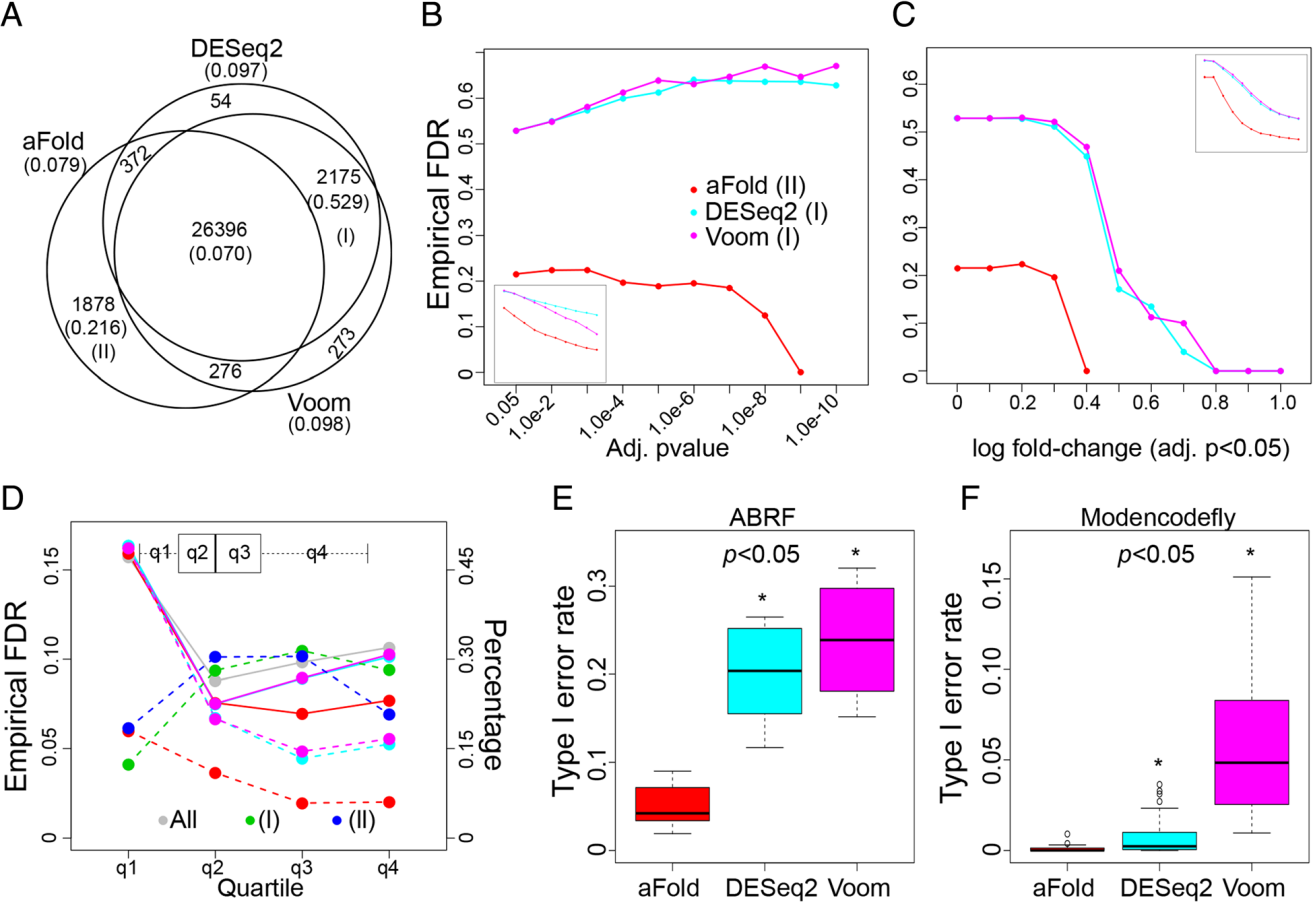
Comparison of methods using real data sets. **a**-**e** Analysis results based on the ABRF data set. **f** Analysis with the modencodefly data set. (**a**) Venn diagram of the number of DE genes identified by the three methods. Numbers in brackets indicate the eFDR. The specific gene sets of aFold alone or DESeq2 and Voom combined are indicated by roman numbers I and II, respectively. **b**-**c** eFDR as a function of different cut-offs of either adjusted *p*-value (**b**) or fold change (**c**) for gene sets I (aFold) and II (DESeq2 and Voom). The two inlets show the results based on all DE genes (rather than the subset of genes). **d** eFDR (left Y axis) and percentage of detected DE genes (right Y axis) for different quartiles of the data (X axis). Solid lines indicate eFDR under adjusted *p*-values of 0.05, dashed lines under adjusted *p*-values of 0.05 and a log fold change of at least 0.5. Red, turquois, and magenta are as in **b** and **c**. Grey line and points show eFDR for all genes (including both DEs and non-DEs). Genes were grouped according to expression (q1, q2, q3 and q4 in boxplot). Lines in blue and green show percentages of detected true DE genes across quartiles for gene set I and II, respectively. **e**-**f** Type I error rates for the ABRF (**e**) and modencodefly (**f**) data sets. Type I error rates are calculated *via* the number of DEs under *p*-value < 0.05 divided by the total number of genes

Another important aim of reliable DE detection is to control the false discovery rate (FDR) and minimize the type I error rate (i.e., the null hypothesis is falsely rejected) while identifying a large number of DE genes [18, 38]. To assess these two aspects, we compared the ability of the alternative approaches to control FDR and type I error rates, using again the ABRF data set and, additionally, the modencodefly data set. Results are summarized in Fig. 7, which are specifically based on fold change related methods: aFold, Voom and DESeq2. Most other methods were already tested on ABSSeq [10].

We first evaluated the three methods using the ABRF data set, based on the same structure as above (e.g., results shown in Fig. 4). Method performance was assessed with the help of empirical FDR (eFDR), which is the ratio between the number of true false positives and the sum of true and false positives (total number of detected DE genes) (Fig. 7a-d). We also investigated the influence of expression levels (Fig. 7d) and additional cut-offs (Fig. 7b-d) on eFDR. The three methods identify similar numbers of DE genes under the adjusted *p*-value of 0.05, whereby Voom reports the largest number (29120), followed by DESeq2 (28997) and aFold (28,922, Fig. 7a). Moreover, when cut-offs for fold change, expression level and adjusted *p*-value are combined, then these three methods report nearly the same number of DE genes, namely 12,976, 14,251 and 14,250 for aFold, DESeq2 and Voom, respectively (84% overall overlap). These results suggest that the above observed differences between aFold and the other two methods result from genes with low expression level and/or fold change, which is consistent with findings from previous studies [8, 10]. As aFold identifies the smallest number of DE genes of the three methods, it also produces a lower overall eFDR (0.079) than both DESeq2 (0.097) and Voom (0.098). This may indicate that aFold is able to control FDR without reducing sensitivity (total number of DE genes) as shown in Fig. 6c and d for simulated data sets.

Interestingly, the genes commonly identified by the three methods retain an eFDR of 0.070, which is close to the used adjusted *p*-value cut-off. The additional difference in identified DE genes may thus be due to model-dependent biases, either as a consequence of the normalization or the statistical approach implemented. In fact, the eFDRs for the method-specific genes are much higher than those for the commonly identified genes. In particular, the genes only revealed by aFold (denoted as the gene subset II) have an eFDR of 0.216, while those jointly identified by DESeq2 and Voom (denoted as the gene subset I) produce an eFDR of 0.529. Note that other subsets were not considered because they included only a small number of genes, which does not permit reliable eFDR calculation. The higher eFDR for gene subset I relative to gene subset II may suggest a larger bias caused by DESeq2 and Voom. Similar results are also observed in the SEQC and MAQC-II data sets (Additional file 1: Figure S8C and D).

Interestingly, when data was normalized by TMM (Voom) or the geometric mean approach (DESeq2), both subsets are reduced (Additional file 1: Figure S8A and B). In this case, only few genes are detected uniquely by aFold, suggesting that aFold retains higher specificity than alternative methods. The subset I is a result of the normalization procedure in aFold (qtotal), which retains low eFDR of 0.216 and supports the efficiency of qtotal normalization. However, it also suggests that genes in subset I actually have comparatively low fold changes. These three DE and normalization methods yield similar results when applied on a data set that contains a small percentage of DE genes (Bottomly data set, Additional file 1: Figure S8 E, F and G).

Our next analysis aimed at reducing eFDR for these two gene subsets by applying more stringent adjusted *p*-value cut-offs (Fig. 7b) or additional fold change cut-offs (Fig. 7c). Both alternatives can improve the overall eFDR (for the entire set of DE genes, inlet figure in Fig. 7b and c). However, eFDR for gene set I is not reduced through adjusted *p*-value cut-offs but rather increases with higher cut-off values. Fold change together with adjusted *p*-value can efficiently decrease eFDR for subset I to a level of 0.05. On the other hand, both cut-offs consistently reduce eFDR of subset II to 0.05 (adjusted *p*-value of 1.0e-9 or 0.05 with log fold-change = 0.4). These results suggest that high eFDR of subset I and II is due to low fold changes (low dispersion).

Since false positives often result from under-estimation of variances (with low fold change but high expression or high fold change but low expression) [8–10], we compared eFDRs across different categories of expression level (four quartiles, Fig. 7d). Indeed, many genes from subset I and II come from the 1st (low expression) and 4th (high expression) quartile (given in light blue and green in Fig. 7d, Y axis on the right side of the panel). Generally, eFDRs at 1st and 4th quartile are higher than 2nd and 3rd for total (grey line). aFold (red line) shows generally lower eFDRs in all quartiles but the 1st one than those obtained for all genes (both DE and non-DE genes, grey line in Fig. 7d, Y axis on left side), whereas DESeq2 (blue line) and Voom (pink line) show a similar pattern than that found for all genes. This observation may suggest that aFold is able to improve eFDR at most of expression levels.

We then assessed use of an additional fold change cut-off of 0.5 (under log2-tranformation). In this case, eFDR reduces to around 0.05 in all quartiles for aFold but only the upper ones (3rd and 4th) for DESeq2 and Voom, which produce no change in the 1st quartile (0.164 to 0.164) and only a slight improvement in the 2nd quartile (0.075 to 0.067). In fact, reducing eFDR for DESeq2 and Voom in 1st quartile to a similar value of 0.05 requires an extremely high log fold change cut-off of 4.0. Such a cut-off additionally decreases the total number of DE genes to 3564 and 3224 for DESeq2 and Voom, respectively. At the same time, applying a log fold change cut-off of 0.5 for aFold still yields a total of 15,339 DE genes. A more efficient way to reduce eFDR at 1st quartile for DESeq2 and Voom is to use a combination of cut-offs for expression level and also *p*-value (eFDR = 0 under logCPM> 0 & adjusted *p*-value < 0.05). These results therefore suggest that aFold is able to control FDR by reducing false positives at all expression level while retaining sensitivity, even when more stringent cut-offs are used.

We next compared the methods in their ability to control type I error rates (i.e., the null hypothesis is falsely rejected and thus results in false positives). We used two gene expression data sets: 1) the ABRF data set as above, including data from the same RNA sample but generated by three different laboratory sites, each with 3 replicates; and 2) the modencodefly data set, which contains data for development processes of the fruitfly *Drosophila melanogaster* [39], with technical replicates ranging from 4 to 6. For the modencodefly data set, we randomly selected 4 replicates for each condition and separated them into two groups, which should thus only be characterized by stochastic variations but not true DE. The results of our analysis are summarized in Fig. 7e (ABRF) and 7F (modencodefly). At the *p*-value cut-off of 0.05, only aFold is able to control the type I error rate around 0.05 for the ABRF data set while the other two methods produce a rate above 0.2. For the modencodefly data set, all three methods are able to control type I error rate around 0.05, but aFold reports the smallest number of false positives (average of 15), followed by DESeq2 (119) and Voom (867). Thus, for both data sets, aFold reduces the type I error rate to a larger extent than the alternative methods (Wilcoxon rank test, *p* < 0.1), consistent with above results for eFDR.

Taken together, the approach implemented in aFold is able to control FDR and type I error rates more effectively than the two tested alternative approaches. Moreover, *p*-values inferred from aFold are directly deduced from and thus monotonically correlated with fold-change, which allows to apply single cut-off values to select candidate DE genes for further analysis. More importantly, aFold also takes into consideration uneven dispersion across expression levels, which avoids possible biases in inferred DE genes due to large fold change at low expression level [10] and thus permits comparable analysis of DE across different types of data distributions (and thus gene expression characteristics).

### Improved visualization of RNA-Seq data

The results of transcriptomic studies are often visualized using a heatmap, which usually takes log fold change as input data to compare the expression difference across treatments or conditions [40, 41]. However, ordinary fold change ignores sample variance, potentially yielding artefactual differences. aFold takes the observed and mean related variance into account during fold change calculation and, thus, it produces more consistent fold change measures across groups. Here we used the ABRF data set to demonstrate how aFold improves the visualization of RNA-Seq data. The ABRF data sets consist of RNA-Seq data from the same RNA samples, measured under two conditions, but processed and analyzed by three different laboratories. The inferred DE variation among lab sites should only result from random or batch effects of (unwanted) environmental or procedural variations, for example due to some differences during library preparation and/or sequencing error. The analysis results indeed identify a high overlap across lab sites of more than 80% for DE genes detected by the three methods.

**Fig. 8:**
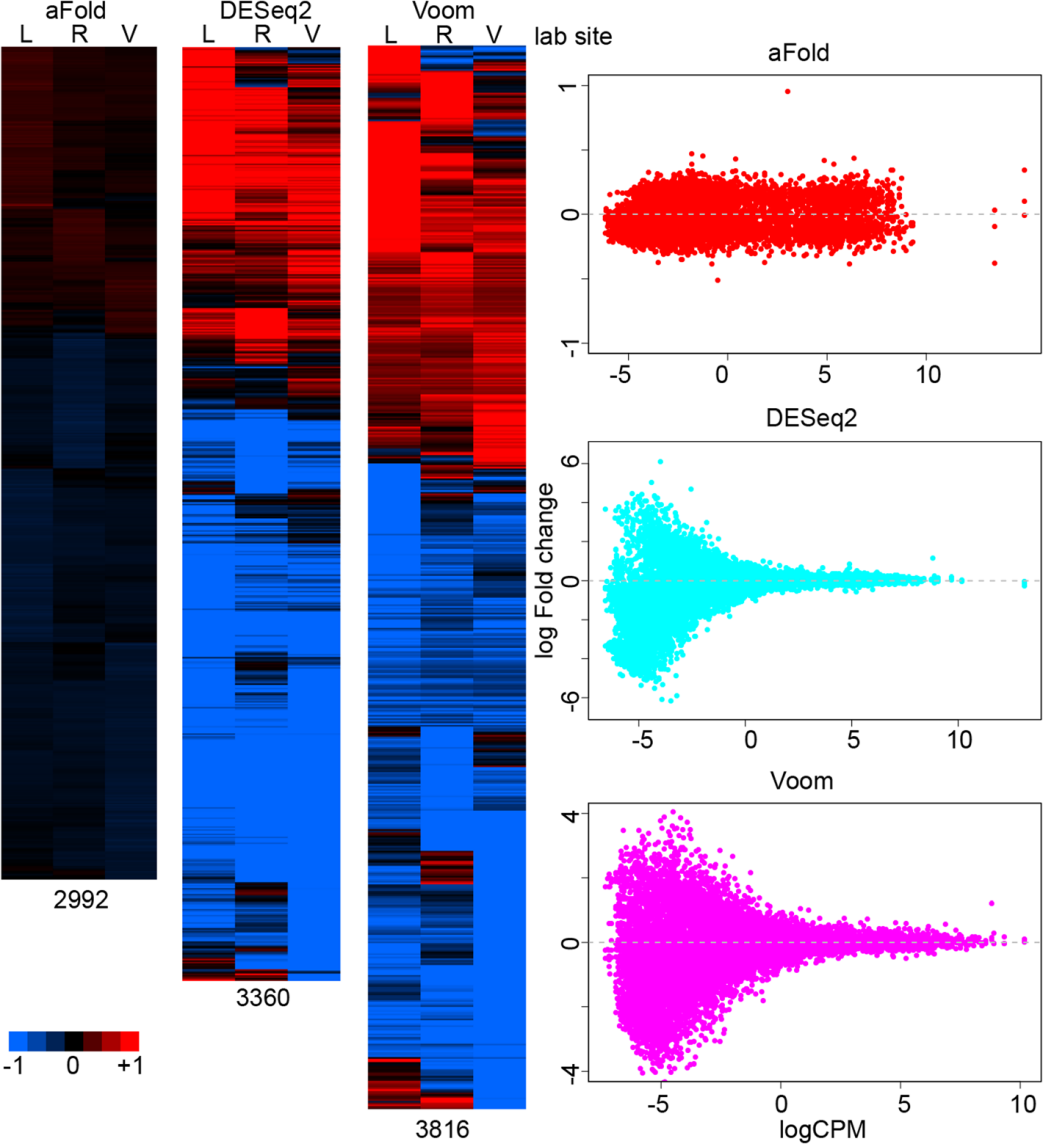
aFold improves visualization of RNA-Seq data. Heatmap of DEs from the same condition but different lab sites that only show significance in one of the lab sites (based on the ABRF data set). Numbers below heatmaps indicate the total number of genes included. Scatter plots show log fold change distribution across expression levels in each heat map. L, R and V stand for lab sites

However, there are still unique genes identified at each lab site by each method, which are most likely caused by variance under-estimation due to limited sample size (*n* = 3). We take genes that show significance at one lab site from each method as unique genes for each method (adjusted *p*-value < 0.05), as illustrated in Fig. 8. aFold identifies the smallest number of genes with unique DE (most of them retain log fold change < 0.5) at only one site but similar pattern across three lab sites (all are up or down-regulation). In contrast, DESeq2 and Voom report many genes that show opposite regulation patterns with high fold change (log fold change > 1), which are likely caused by high dispersion across samples and lab sites.

Fold change measures reported by DESeq2 and Voom are unable to capture the magnitude of expression differences and therefore might result in unreal opposite regulation patterns. Indeed, over 75% of genes in each unique set show very low expression (logCPM < 0). These genes also often exhibit high variance combined with high fold change (Fig. 8, scatter plot) [3, 4, 10], thus requiring shrinkage of fold change or additional filtering of expression level to reduce false positives [6, 8–10]. The difficulty here is that there is no universal cut-off value for expression levels because reliability additionally depends on sample size (e.g., large sample size can enhance reliable variance estimation in highly dispersed data and thus also reduces error rates). Here, we demonstrate that aFold is able to accurately estimate fold change by taking into account variance. Thus, aFold improves the visualization of expression data by reducing DE variation, which in turn will facilitate pattern discovery (clustering) and gene set enrichment analyses [41].

## Conclusions

Here, we introduce a new approach for normalization and DE analysis of RNA-Seq data. The new normalization procedure included in the package, qtotal, adjusts for the influence of the number of DE genes on the overall read count distribution and accurately approximates the true sequence depth. Qtotal can also be combined with different RNA-Seq analysis methods outside of aFold. It results in DE identification that is at least as good as and often better than those produced with alternative normalization procedures, especially in case of asymmetrical distrbutions of up- and down-regulated genes. The new fold change inference and analysis method, the aFold DE analysis algorithm, is distinct from other current methods, because it uses polynomial uncertainty modelling to infer fold changes and considers variance in read count data across genes and treatment groups. It thus permits reliable fold-change comparisons across genes, which will enhance correct ranking of genes for selection of candidates for subsequent analysis and gene set enrichment analysis [41]. Using real and simulated data sets, we demonstrate that aFold is at least as efficient as and often better in discriminating DE and non-DE, especially in the presence of outliers or biased DE distrubutions. Our statistical framework shows high power to control FDR and type I error rate across expression levels. Based on our analyses, we conclude that the aFold package represents a highly efficient novel tool for RNA-Seq data normalization, fold change estimation, and identification of significant DE across a wide range of conditions. It may help the experimentalist to avoid an arbitrary choice of cut-off thresholds and may enhance subsequent downstream functional analyses.

## Materials and methods

### Datasets

We used two main types of data sets: simulated and real data. They are described in detail in the supplemnetary methods in Additional file 1. An overiew of the real data sets is given in Table 1, including the average total number of read count, the number of genes, sample size and the average number of DE genes. Genes with zero counts in all samples were filtered out for analysis.

### Normalization

The procedure underlying the new qtotal normalization method is provided in detail in the supplementary methods in Additional file 1. The alternative, previously available normalization methods were all used with default settings (voom and TMM for Voom and edgeR, geometry mean for DESeq2, qtotal for ABSSeq, quartile for baySeq, total for ROTS).

### Moderating uncertainty of read count in the aFold approach

Due to biological and/or other sources of variance, the observed expression value for the i^th^ gene *g*_*i*_ is given as the mean *μ*_*i*_ with uncertainty *ε*_*i*_.

1 $$ {c}_i={\mu}_i+{\varepsilon}_i $$

In practice, the uncertainty is represented as the standard deviation (SD) of samples if the SD is independent of the mean. However, In RNA-Seq data or microarray data, the SD is not independent of *μ*_*i*_ and could be generally written as

2 $$ {\sigma}_i={a}_i{\mu}_i\kern0.75em {a}_i>0 $$

where *a*_*i*_ is the coefficient that stands for the mean-variance relationship of the i^th^ gene. This implies that there is propagation of error (uncertainty) in measurement of SD based on *μ*_*i*_. Therefore, an accurate reads uncertainty measurement should also include the propagation of error from (2). In theory, the propagation uncertainty of SD can be written as

3 $$ {\varepsilon}_{i,s}={a}_i SD\kern0.5em \left({g}_i\right)={a}_i{s}_i $$

where *s*_*i*_ is the sample SD of *g*_*i*_. Thus, the uncertainty of read counts for each gene becomes

4 $$ {\varepsilon}_i={s}_i+{\varepsilon}_{i,s}={s}_i+{a}_i{s}_i $$

*a*_*i*_ in (3) actually serves as the CV as

5 $$ {a}_i=\frac{\sigma_i}{\mu_i}\approx \frac{s_i}{\mu_i} $$

The uncertainty of *g*_*i*_ becomes a polynomial function of sample SD *s*_*i*_

6 $$ {\varepsilon}_i={s}_i+\frac{s_i^2}{\mu_i} $$

*ε*_*i*_ is then used to moderate the ordinary fold change (see Additional files for details). A more detailed description of the new analysis method and also its evaluation in comparison with available approaches is given in the supplementary methods in Additional file 1. An illustration of aFold modelling is provided in Additional file 1: Figure S9.

### Implementation

aFold has been implemented and integrated in the software package ABSSeq for the cross-platform environment R, available through the R Core Team [42]. aFold is released under the GPL-3 license as part of the Bioconductor project [32] at URL: http:// bioconductor.org/packages/devel/bioc/html/ABSSeq.html.

### Software tools

The figures in this study have been plotted using R.

## Additional file

Additional file 1: Supplementary Methods, Figures and Tables. (PDF 2321 kb)

## Abbreviations

AUC: Area under curve
DE: Differential expression
FC: Fold change
FDR: False discovery rate
FPR: False positive rate
logFC: log2 of fold change
NB: Negative binomial
RNA-Seq: (high-throughput) sequencing of RNA
ROC: Receiver operating characteristic
SEQC: Sequencing Quality Control
TPR: True positive rate
