# Supplementary material for: aFold – using polynomial uncertainty modelling for differential gene expression estimation from RNA sequencing data
Source: BMC Genomics. 2019 May 10;20:364. doi: 10.1186/s12864-019-5686-1 (PMC6509820; doi:10.1186/s12864-019-5686-1)
Supplement: Supplementary file 1 — Supplementary Methods, Figures and Tables. (PDF 2321 kb) [file 12864_2019_5686_MOESM1_ESM.pdf]

Supplementary Methods

Datasets

Results of this study are based on two main types of data sets: simulated and real data. The simulated data sets are derived from the study of Soneson et al. [1], which simulated read count data for 12,500 genes from a negative binomial (NB) distribution with mean and variances from Pickrell's RNA-Seq dataset [2]. Pickrell's data set consists of 69 lymphoblastoid human cell lines derived from unrelated Nigerian individuals. The simulated data is generated under two conditions: NB and NB with random outliers (denoted by R). Each set includes 10 independently repeated simulations of two treatment groups and different replicate sample sizes of 2, 5 or 10 for each group. Outliers were introduced into the NB distributed data by multiplying a randomly generated factor between 5 and 10 with the read count of all genes in all groups obtained through random sampling with a probability of 0.05.

In addition, seven real data sets were used to assess the performance of DE inference methods (Table 1). The first four data sets are based on replicated RNA samples of the human whole body (UHR) and brain (BHR) [3, 4]: ABRF, MAQC-II, SEQC, and PrimePCR (qRT-PCR validated data set to define true DE). The ABRF data set refers to the Association of Biomolecular Resource Facilities next-generation sequencing (ABRF-NGS) study, which assessed RNA-Seq data variation across laboratory sites and platforms [5] . Here we use

data from two samples generated via a ribo-depleted protocol, namely RNA from cancer cell lines and also RNA from pooled normal human brain tissues. We thus exclude data from mixtures of these samples and that based on other protocols. The raw data and counts tables are available at the Gene Expression Omnibus database under accession number GSE48035. The considered RNA-Seq data compares two conditions (UHR and BHR), whereby the same RNA samples are analyzed in three different laboratories. Any variation between these laboratories should not be due to relevant biological differences, but result from variations across sites in environmental and also procedural factors. The MAQC-II data set consists of seven replicates for each condition and is generated by the MicroArray Quality Control (MAQC) study to evaluate the performance of different gene expression analysis methods [6]. The raw data of MAQC-II are available from the NCBI SRA database under SRA010153 and counts table is downloaded from http://bowtie-bio.sourceforge.net/recount/ [7]. The SEQC data set consists of five replicates and is generated by Sequencing Quality Control (SEQC) study available under GSE49712. The ABRF are aligned to External RNA Control Consortium (ERCC) transcript using STAR 2.5.3a [8] to obtain the information for ERCC. The PrimePCR data set is based on the PrimePCR approach of qRT-PCR and includes more than 20,000 validated DE genes from SEQC (MAQC III), available under GSE56457. The SEQC data set was additionally used to assess the influence of sequencing depth on method performance. We derived five data sets, which only had 10%,

20%, 30%, 40%, and 50% of the read numbers of the original SEQC data set, followed by their analysis with the various DE methods and normalization procedures.

Three additional real data sets were downloaded from http://bowtie-bio.sourceforge.net/recount/ [7]. The first of these is the modencodefly data set from the modENCODE project [9], which assesses gene expression during the development of *Drosophila melanogaster* [10], covering 30 distinct developmental stages. Each of the stages consists of 4 up to 6 technical replicates, which provides an opportunity to construct subgroups per developmental stage to study stochastic variations but not true DE. We accordingly subsampled from each stage to construct a 2:2 pairwise study.

The next real data set is the HapMap-CEU data set [11], which includes 41 samples based on immortalized B-cells from 41 unrelated CEPH grandparents. It contains a relatively large sample size (17 female samples and 24 male samples) and high variations in read count due to genetic diversity. It is well-studied and useful for measuring the ability of DE detection models on large samples and variations [12-14].

We also considered the Bottomly data set, which is from a study that characterized transcriptomic differences between two inbred mouse strains (C57BL/6J and DBA/2J) with 10 and 11 replicates each, respectively [15]. We filtered out genes with zero read count across samples before analysis.

The basic statistics for all real data sets are summarized in Table 1 of the main text, including the average total number of read count, the number of present genes, sample size and the average number of DE genes. Genes with zero counts in all samples are filtered out for analysis

Normalization

Read count of RNA-Seq data requires normalization before DE inference in order to reduce possible biases from sequence depth, library preparation or even analysis in sequencing lanes [16, 17]. Current approaches for normalization rely on the assumption that the majority of genes (or at least those with high expression) are not DE and/or that the distribution of up- and down-regulated DEs is symetrical. These assumptions might not be valid under certain biological processes, such as development or aging, when gene expression shows dramatic biological variation or samples with unsymmetrical DE. In contrast to current normalization approaches, we assess the overall distribution of DE across samples and then use it to estimate the true library size ratio χ (i.e., ratio between total number of reads from two samples) from the observed library size ratio χ_o between two samples (e.g., sample A and B). χ_o is a function of χ and influence of DE d as

$$\log(\chi_o) = \log(\chi) + d \tag{1}$$

where d could be positive or negative depending on whether DE is mainly influenced from up- or down-regulated genes, respectively. Biological variation results in log fold changes under a zero centered normal distribution with standard deviation (SD) σ. DE scales σ as a half normal distribution with parameters τ_r and τ_l (e.g., up and down-regulation in A for two half normal distributions, respectively). In this case, the influence of DE on the log library size ratio for A (d_A, vs. B) or B (d_B, vs. A) is a result of τ_r and τ_l and can be defined as

$$\begin{cases} d_A = \alpha_A \beta \tau_r \sigma - (1-\alpha_A)\beta\tau_l\sigma \\ d_B = \alpha_B \beta \tau_l \sigma - (1-\alpha_B)\beta\tau_r\sigma \end{cases} \quad \alpha \in [0,1] \; \beta = \sqrt{\frac{2}{\pi}} \;\; \tau \geq 1 \tag{2}$$

where α is the probability of a gene to be up-regulated in the current sample and $\beta\sigma$ is the mean of the half normal distribution. While α for the entire data should be around 0.5 (equal chance for up- or down-regulation), the observed library size ratio between A and B could be thus approximately represented as

$$\log(\chi_o) \approx \log(\chi) + \beta\sigma(\tau_r - \tau_l) \tag{3}$$

Under (1), the mean ratio of read count between A and B for any subsets in A and B ($\chi_{\{A\}}$ and $\chi_{\{B\}}$) can be written as

$$\begin{cases} \log(\chi_{\{A\}}) = \log(\chi) + d_{\{A\}} \\ \log(\chi_{\{B\}}) = \log(\chi) - d_{\{B\}} \end{cases} \tag{4}$$

where $\{A \| B\}$ indicates a subset of read counts from sample A or B, respectively. Therefore, from (4) we obtain

$$\log(\chi_{\{A\}}) - \log(\chi_{\{B\}}) = d_{\{A\}} + d_{\{B\}} = (\alpha_{\{A\}} + \alpha_{\{B\}} - 1)\beta\sigma(\tau_r + \tau_l) \tag{5}$$

Since up-regulation increases gene expression and thus top-ranked genes are more likely up-regulated (or non-DE), (i.e., $\alpha_{\{A\}\&\{B\}} \rightarrow 1$ in (5)), (5) for the highly expressed subsets (e.g., a large quantile $q_0 = 0.95$ after ranking via reads count) can be approximated by

$$\log(\chi_{\{A\}}) - \log(\chi_{\{B\}}) \approx \beta\sigma(\tau_r + \tau_l) \quad \{A||B\} = c_{\{A||B\}} \geq quantile(c_{A||B}, q_0) \qquad (6)$$

where c indicates read count. While the SD across $\log(c_{A||B})$ (or CV across $c_{A||B}$) is determined by biological variation σ, it is also scaled by DE with τ_r and τ_l as

$$sd = sd_0 \tau_r^{\lambda} \tau_l^{-\lambda} \quad \lambda \in [-1,1] \qquad (7)$$

where sd and sd_0 indicates SDs after and before DE, respectively. λ is the coefficient of $\tau_{r||l}$ on sd_0, which could be increased ($\lambda > 0$) or decreased ($\lambda < 0$) by either up or down-regulation (i.e., both can increase or decrease the difference across read counts). λ thus has opposite effects for up and down-regulated DE on sd_0 and ranges in value from -1 to 1. As a result, the maximum or minimum of sd / sd_0 respectively approaches τ_l / τ_r or τ_r / τ_l when $|\lambda| \rightarrow 1$. Biological varation σ (or sd_0) is determined by expression level [12, 14] (i.e, quantile q of $c_{A||B}$). For the same q (with similar expression level), we assume that subsets of $\log(c_A)$ and $\log(c_B)$ preserve sd_A and sd_B from the same sd_0. The sd / sd_0 ratio could be thus obtained from $sd_{A||B}$ at q as

$$\frac{sd}{sd_0} = \frac{sd_{A|q}}{sd_{B|q}} \qquad (8)$$

Based on this logic, we apply a sliding quantile window on $\log(c_{A\|B})$ to obtain a maximum or minimum of the SD ratio as the estimation of τ_l / τ_r, that is

$$\frac{\tau_l}{\tau_r} = \begin{cases} r_{mx} = \max(\gamma_j) \text{ if } \tau_l > \tau_r \\ r_{mi} = \min(\gamma_j) \text{ if } \tau_l < \tau_r \end{cases} \quad \gamma_j = \frac{sd_{A|q=j}}{sd_{B|q=j}} \; j \in [0, 1-w] \tag{9}$$

where *j* and w are the start and size of sliding window, respectively. A sliding window with small size (e.g, $w = 0.05$) will maximize the influence of τ on sd_0 (i.e., $|\lambda| \to 1$, up- or down-regulation has the same effect on all genes in the subset). The true library size ratio can be estimated from (4), (6) and (9). However, it is impossible to determine whether $\tau_r < \tau_l$ or $\tau_r > \tau_l$ due to the unknown direction of λ. Notably, an opposite choice of τ_l / τ_r (e.g., r_{mi} under $\tau_l > \tau_r$) under (9) will result in an estimation of τ_r as τ_l. As a consequence, $\log(\hat{\chi})$ will become $\log(\chi) + \beta\sigma(\tau_r - \tau_l)$ under (4) and (6), which is close to the observed size ratio χ_o (3). This suggests that we can obtain a more accurate $\hat{\chi}$ by

$$\hat{\chi} = \begin{cases} \hat{\chi}_{r_{mx}} \text{ if } r_d \geq 0 \\ \hat{\chi}_{r_{mi}} \text{ if } r_d < 0 \end{cases} \quad r_d = |\log(\frac{\hat{\chi}_{r_{mx}}}{\chi_o})| - |\log(\frac{\hat{\chi}_{r_{mi}}}{\chi_o})| \tag{10}$$

In practice, we select one sample (default is the one with the largest number of read counts) as control and then apply this procedure on all samples to obtain the size factor for normalization. This procedure is implemented as *qtotal* in ABSSeq and set as default normalization procedure for aFold.

For the additional DE analysis methods, we used the default normalization procedures (voom and TMM for Voom and edgeR, geometry mean for DESeq2, qtotal for ABSSeq, quartile for baySeq, total for ROTS).

Outlier detection

Outliers influence DE detection through shifting both mean and variance [13, 14], which thus needs to be corrected for. Here we integrate the procedure from our previous ABSSeq approach into aFold, which utilizes the median absolute deviation (MAD) to detect the outliers in log-transformed read count and shrink the read count of outliers toward median of read count from one condition.

Moderating uncertainty of read count

Due to biological and/or other sources of variance, the observed expression value for the ith gene g_i is given as the mean μ_i with uncertainty ε_i.

$$c_i = \mu_i + \varepsilon_i \tag{11}$$

In practice, the uncertainty is represented as the standard deviation (SD) of samples if the SD is independent of the mean. However, in RNA-Seq data or microarray data, the SD is not independent of μ_i and could be generally written as

$$\sigma_i = a_i \mu_i \quad a_i > 0 \tag{12}$$

where a_i is the coefficient that describes the mean-variance relationship of the ith gene. This implies that there is propagation of error (uncertainty) in measurement of SD based on μ_i. Therefore, an accurate reads uncertainty measurement should also include the propagation of error from (12). In theory, the propagation uncertainty of SD can be written as

$$\varepsilon_{i,s} = a_i SD(g_i) = a_i s_i \tag{13}$$

where s_i is the sample SD of g_i. Thus, the uncertainty of read counts for each gene becomes

$$\varepsilon_i = s_i + \varepsilon_{i,s} = s_i + a_i s_i \tag{14}$$

a_i in (13) actually serves as the CV as

$$a_i = \frac{\sigma_i}{\mu_i} \approx \frac{s_i}{\mu_i} \tag{15}$$

The uncertainty of g_i becomes a polynomial function of sample SD s_i

$$\varepsilon_i = s_i + \frac{s_i^2}{\mu_i} \tag{16}$$

In addition to the observed variance, there are still hidden variances upon expression levels, which are usually described as

$$\omega_i^2 = \mu_i \tag{17}$$

which is dominated by mean read count of each gene [14]. As a result, the uncertainty from expression level becomes

$$\hat{\varepsilon}_i = \omega_i = \sqrt{\mu_i} \tag{18}$$

We leave out the second term of the polynomial function of (16) in (18) because

ω_i is the expected SD for each gene and contains no propagation error. Since ω_i represents SD from limited samples in practice, $\hat{\varepsilon}_i$ actually sums up uncertainty across samples and thus requires moderation of the sample size according to the central limit theorem as

$$\hat{\varepsilon}_i = \frac{\omega_i}{m_i} = \frac{\sqrt{\mu_i}}{m_i} \qquad (19)$$

where m_i is defined as the effective sample size for each gene. We use the effective sample size instead of the real sample size in (19) to capture the data structure (i.e, overal dispersion of CVs). According to G.4.2 in [18], a global effective sample size (effective degrees of freedom) can be obtained via

$$m = \frac{mean(\overline{v}_i)^2}{\mathrm{var}(\overline{v}_i)} \quad \overline{v}_i = \frac{s_i}{\mu_i + \varepsilon_i} \qquad (20)$$

Instead of using original CVs, *m* is calculated from moderating CVs, which retains information of uncertainty of μ_i and is more stable (Figure S9A), thus avoiding underestimation of *m*. The effective sample size m_i actually varies across expression levels since the biological variation is more difficult to capture at low than high expression levels. We thus assume that the genes with highest expression retain m as m_i while the remaining genes have a decreasing m_i which can be written as

$$m_i = \frac{k\overline{v}_0^2}{\tilde{v}_i^2} \quad \tilde{v}_i = f(\overline{v}_i) \; \overline{v}_0 = \max(v_0, \min(\frac{\omega_i}{\mu_i})) \qquad (21)$$

Where k is the coefficient factor for expression level, $\tilde{v}_i$ is the smoothed CV by the *locfit* package from R [19], f stands for the smoothing function and v_0 is prior value that could be provided by users to avoid over-estimation of m_i

(default is 0.05). We use $\bar{v}_i^2/\bar{v}_0^2$ instead of $\bar{v}_i/\bar{v}_0$ because *k* is proportional to $\bar{v}_i^2$ as in (21). The final uncertainty $\bar{\varepsilon}_i$ for the ith gene is then called as

$$\bar{\varepsilon}_i = \varepsilon_i + \hat{\varepsilon}_i = \varepsilon_i + \frac{\sqrt{\mu_i}}{m_i} \qquad (22)$$

Moderating fold change by uncertainty of read count

In our previous study [13], we show that the log fold change can be described as

$$lfc_i = \log(\frac{c_i}{c_i - \Delta_i}) \;\; c_i = \max(\mu_{iA}, \mu_{iB}) \;\; \Delta_i = |\mu_{iA} - \mu_{iB}| \qquad (23)$$

This relationship specifies that log fold change depends on the expression level and the mean read count difference between two conditions (denoted as A and B). Under (23), the same mean difference Δ_i refers to larger log fold change at lower expression level c_i. The uncertainty (as variance) could be treated as unobserved read count, thus the actual or robust fold change can then be written as

$$lfc_i = \log(\frac{\hat{c}_i}{\hat{c}_i - \Delta_i}) \;\; \hat{c}_i = c_i + \bar{\varepsilon}_i \qquad (24)$$

The fold change is thus shrunk toward 0 according to uncertainty or variance. As a result, the fold change from (24) presents a robust way of measuring differential expression since it fully accounts for the mean and variance of expression values. We thus term this inference procedure the accurate fold change (aFold) approach.

Determination of the cut-off of aFold

While the ordinary log fold changes usually follows a normal distribution with zero mean [14, 20, 21], aFold also has a zero-centered normal distribution (Figure S9B, HapMap-CEU). Therefore, the cut-off (significance threshold) of aFold can be determined by estimating the SD of the zero-centered normal distribution. Notably, the aFold calculation approach is equivalent to adding the pseudocounts ($\bar{\varepsilon}_i$ from (22)) to read count. This has no influence on read count variance, but stabilizes the CV (variance stabilization, inlets in Figure S9A, S9C and S9D). Based on this procedure (i.e., adding $\bar{\varepsilon}_i$), we obtain for each data set a general CV for the count level or SD for log transformation of counts (also as the SD for aFold). We can next calculate the general SD under log transformation via moments estimation as

$$\sigma = mean(s_{\log}) / \sqrt{n-1} \tag{25}$$

σ from (25) fits the distribution of aFold very well (Figure S9B, red line). Then the p-value of each aFold is generated via the normal distribution as

$$p = pnorm(lfc_i, 0, \sigma) \tag{26}$$

After an adjustment of multiple testing (i.e, Benjamini-Hochberg in default), a data-specific aFold cut-off is obtained in consideration of the significance level. The aFold model is here developed for simple designs. It is possible to adjust it for complex experimental designs with the help of linear models from limma

[22]. Such more complex designs will be assessed and implemented in the future.

Supplementary Tables

Table S1. Comparison of AUCs in Figure 1. Pvalues are calculated via a two sample one side z-test.

Dataset	qtotal vs.	Z-score	Adjusted pvalue
ABRFAB	TMM	10.79	1.40E-26
	total	2.48	4.56E-02
	quartile	0.15	1.00E+00
	geometric	9.44	1.29E-20
	cqn	5.09	1.24E-06
	MedpgQ2	6.80	3.60E-11
	UQpgQ2	8.60	2.81E-17
SEQC	TMM	9.97	7.17E-23
	total	6.80	3.70E-11
	quartile	7.39	5.03E-13
	geometric	12.81	5.37E-37
	cqn	10.60	1.09E-25
	MedpgQ2	8.92	1.67E-18
	UQpgQ2	7.98	5.22E-15
MAQC-II	TMM	7.49	2.42E-13
	total	-0.03	1.00E+00
	quartile	3.80	5.03E-04
	geometric	8.71	1.05E-17
	cqn	10.60	1.01E-25
	MedpgQ2	3.54	1.40E-03
	UQpgQ2	5.25	5.27E-07

Table S2. Comparison of AUCs in Figure 4. Pvalues are calculated via a two sample one side z-test.

Dataset	Comparison		Z-score	Adjusted pvalue
ABRFAB	aFold vs.	DESeq2	18.62	1.76E-76
		edgeR	17.12	8.92E-65
		Voom	19.75	6.45E-86
		baySeq	1.93	4.31E-01
		ABSSeq	0.03	1.00E+00
		ROTS	5.46	3.90E-07
	DESeq2	DESeq2-qtotal	14.19	8.97E-45
	edgeR	edgeR-qtotal	15.79	2.90E-55
	Voom	Voom-qtotal	19.75	6.63E-86
	baySeq	baySeq-qtotal	0.33	1.00E+00
	ROTS	ROTS-qtotal	0.35	1.00E+00
SEQC	aFold vs.	DESeq2	16.29	9.67E-59
		edgeR	10.11	4.08E-23
		Voom	13.56	5.50E-41
		baySeq	8.75	1.71E-17
		ABSSeq	1.25	1.00E+00
		ROTS	1.06	1.00E+00
	DESeq2	DESeq2-qtotal	12.54	3.75E-35
	edgeR	edgeR-qtotal	10.30	5.40E-24
	Voom	Voom-qtotal	14.69	5.64E-48
	baySeq	baySeq-qtotal	7.15	7.00E-12
	ROTS	ROTS-qtotal	7.07	1.28E-11
MAQC-II	aFold vs.	DESeq2	13.18	8.65E-39
		edgeR	11.36	5.37E-29
		Voom	11.43	2.29E-29
		baySeq	6.26	3.12E-09
		ABSSeq	1.64	8.15E-01
		ROTS	3.74	1.50E-03
	DESeq2	DESeq2-qtotal	8.66	3.67E-17
	edgeR	edgeR-qtotal	7.34	1.75E-12
	Voom	Voom-qtotal	9.36	6.34E-20
	baySeq	baySeq-qtotal	4.81	1.23E-05
	ROTS	ROTS-qtotal	0.03	1.00E+00

Table S3. List of the seven sex-related genes for HapMap analysis.

Ensemble ID	Gene_name	Chromosome	Start	End
ENSG00000157828	RPS4Y2	Y	22918050	22942918
ENSG00000099749	CYorf15A	Y	21729235	21752309
ENSG00000129824	RPS4Y1	Y	2709527	2734997
ENSG00000154620	TMSB4Y	Y	15815447	15817904
ENSG00000198692	EIF1AY	Y	22737611	22755040
ENSG00000183878	UTY	Y	15360259	15592553
ENSG00000006757	PNPLA4	X	7866288	7895780

The seven genes are either located on the Y or X chromosomes.

Supplementary Figures

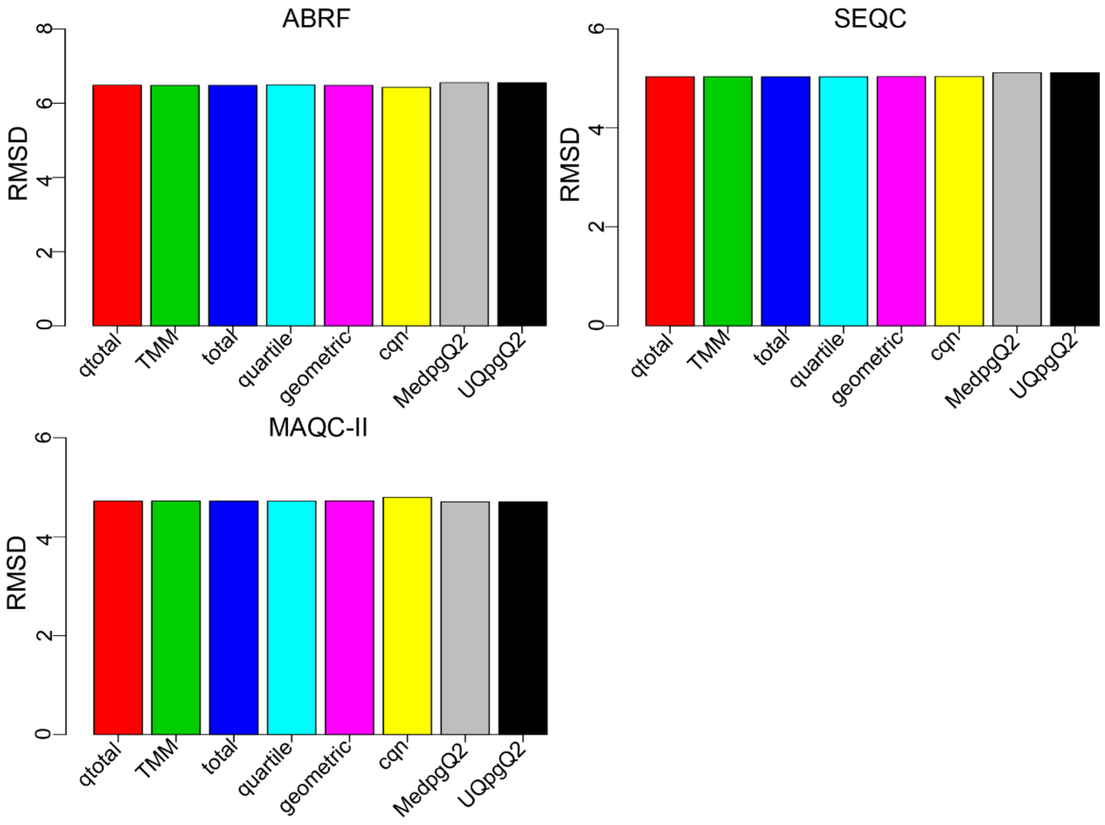

Figure S1. RMSD (root mean square deviation) correlation between log2 fold changes from PrimePCR and RNA-Seq data: ABRF, SEQC and MAQC-II.

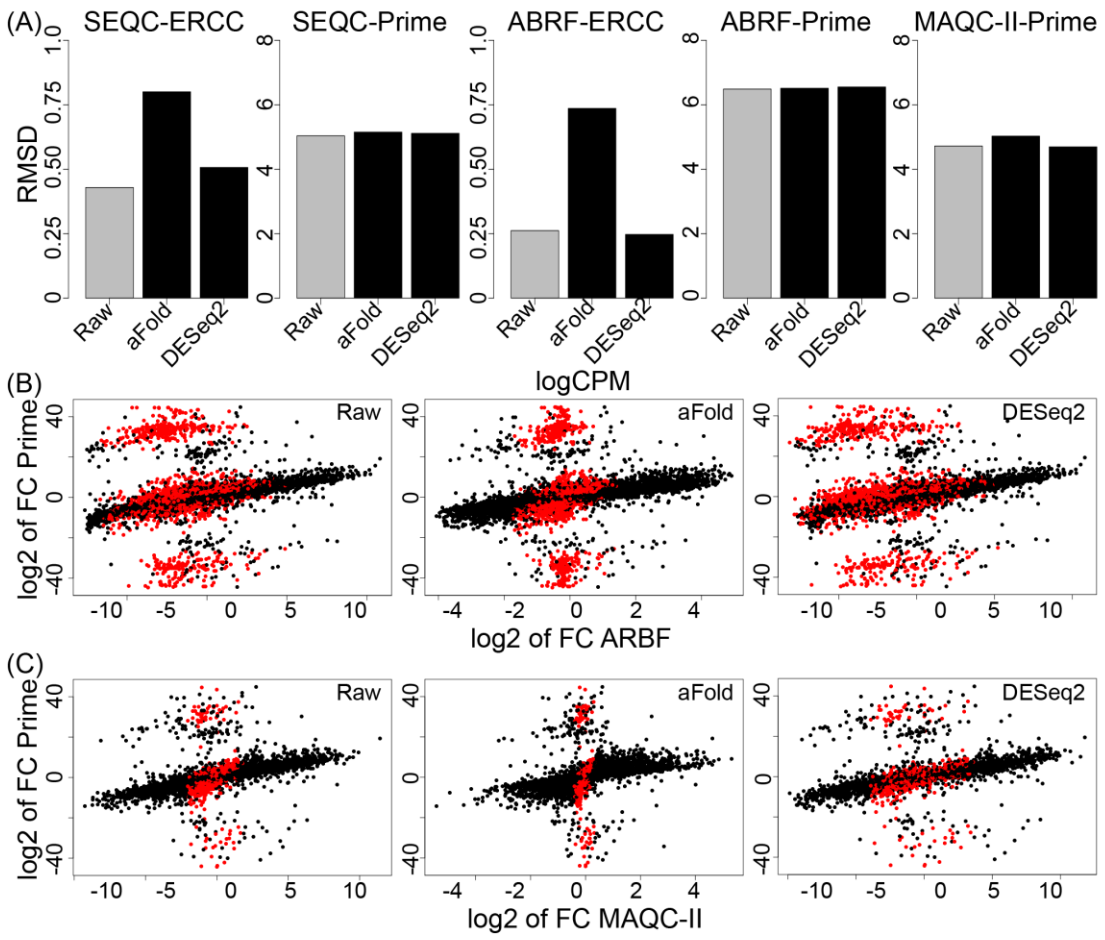

Figure S2. Correlation between true and estimated fold changes. (A) RMSD (root mean square deviation) correlation between log2 fold changes from ERCC, PrimePCR (labeled as Prime) and RNA-Seq data: ABRF, SEQC and MAQC-II. Scatter plot of fold changes from PrimePCR (y-axis) and RNA-Seq: (B) ABRF and (C) MAQC-II. Lowly expressed genes (logCPM < 1) are represented by red points.

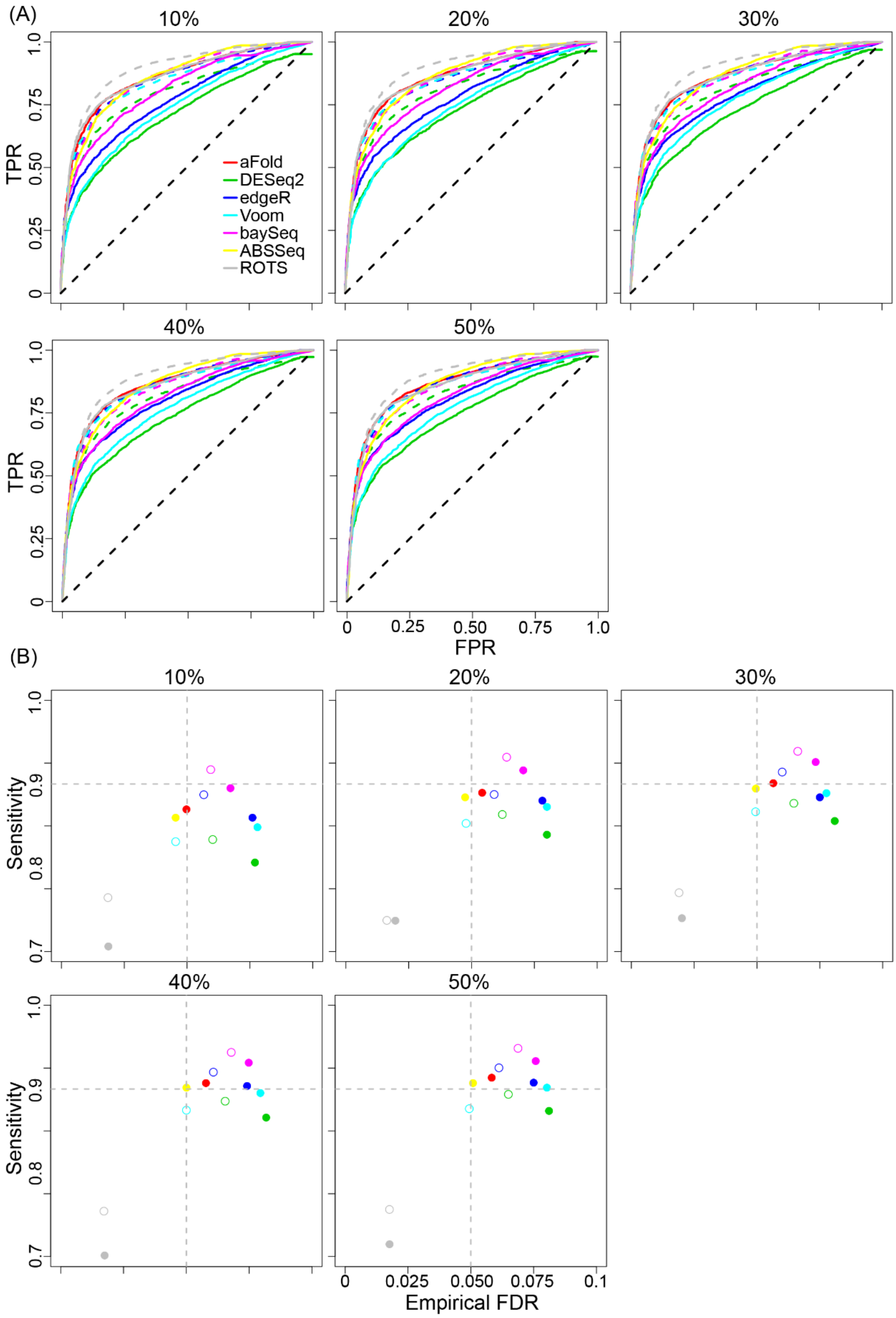

Figure S3. Assessment of method performance for different sequencing depths of the SEQC data set. The different sequencing depths were

generated with SAMtools [23], using either 10%, 20%, 30%, 40%, or 50% of the original number of reads. (A) ROC analysis. Solid lines show the results for the RNA-Seq methods with their integrated normalization procedures. Dashed lines (except diagonal) show the results under qtotal for all methods except for the two methods, aFold and ABSSeq, which use qtotal as default and are thus shown as solid lines. qtotal improves the performance of most methods. (B) Sensitivity analysis. Sensitivity is calculated as the ratio between the number of true DE genes under adjusted pvalue < 0.05 and the total number of true DE genes, inferred from PrimePCR. The empirical false discovery rate (eFDR) is calculated as FPs/(TPs+FPs) under adjusted pvalue < 0.05. qtotal improves either eFDR or sensitivity or both when applied with the tested DE methods. Filled and open circles indicate results for methods with their default normalization approach and qtotal, respectively.

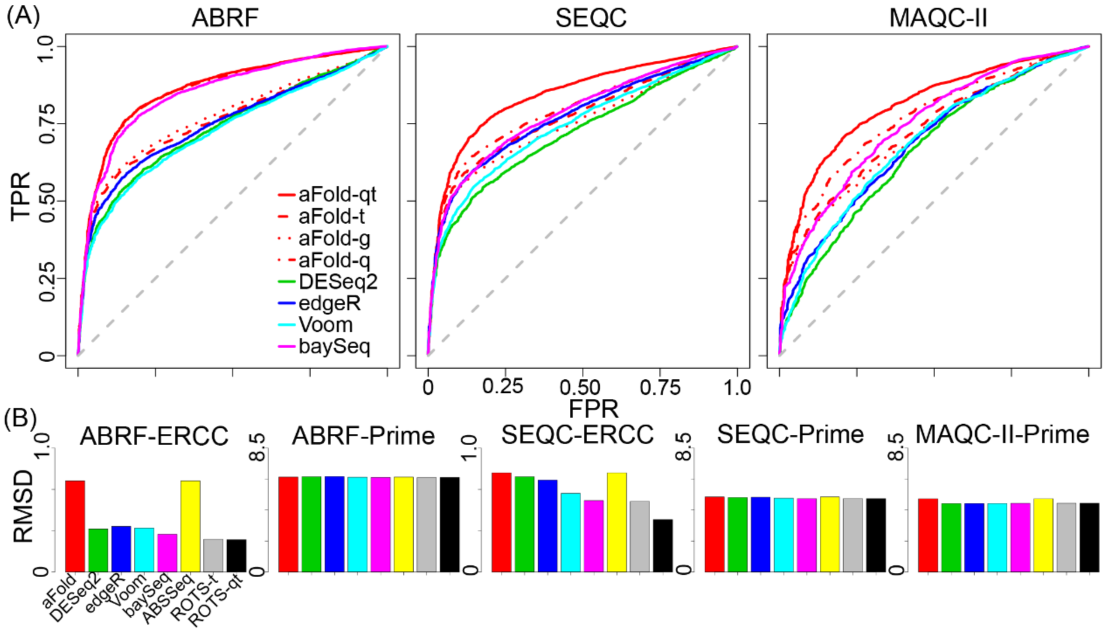

Figure S4. Additional analysis using the qRT-PCR validated data sets. (A) ROC analysis. Results of aFold are shown with different normalization methods: TMM (labeled as aFold-t), geometric (aFold-g), quartile (aFold-q) and qtotal (aFold-qt). (B) RMSD (root mean square deviation) correlation between log2 fold changes from ERCC, PrimePCR (labeled as Prime) and RNA-Seq data: ABRF, SEQC and MAQC-II as well as different DE methods.

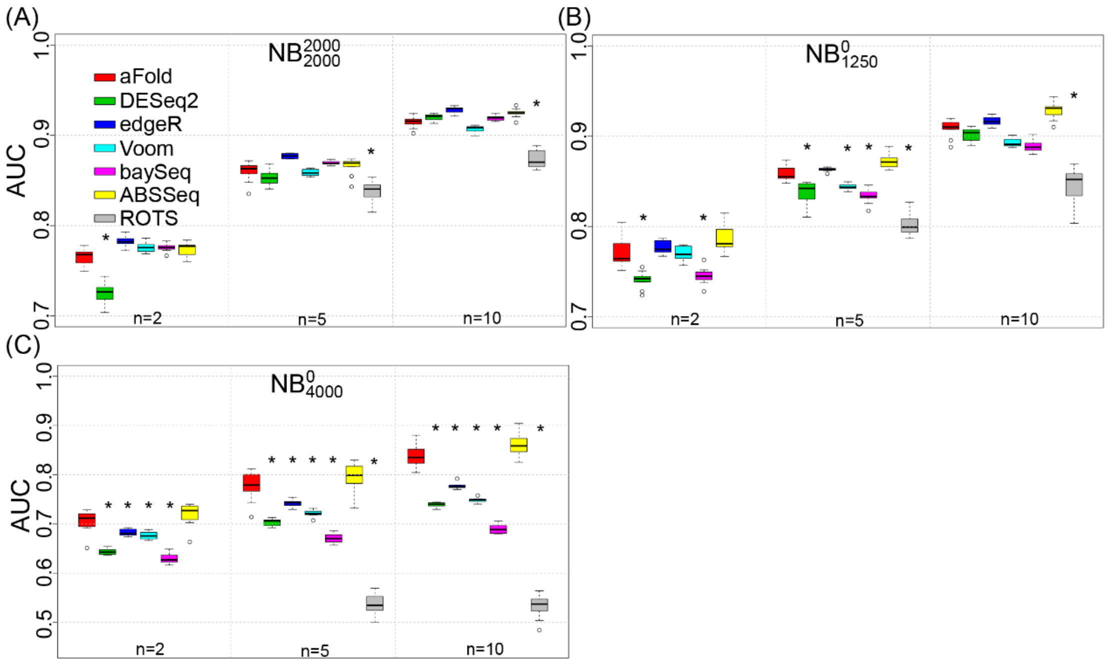

Figure S5. Additional AUC analysis on simulated data sets. Asterisk indicates a statistically significant difference in AUC between aFold and any of the other methods.

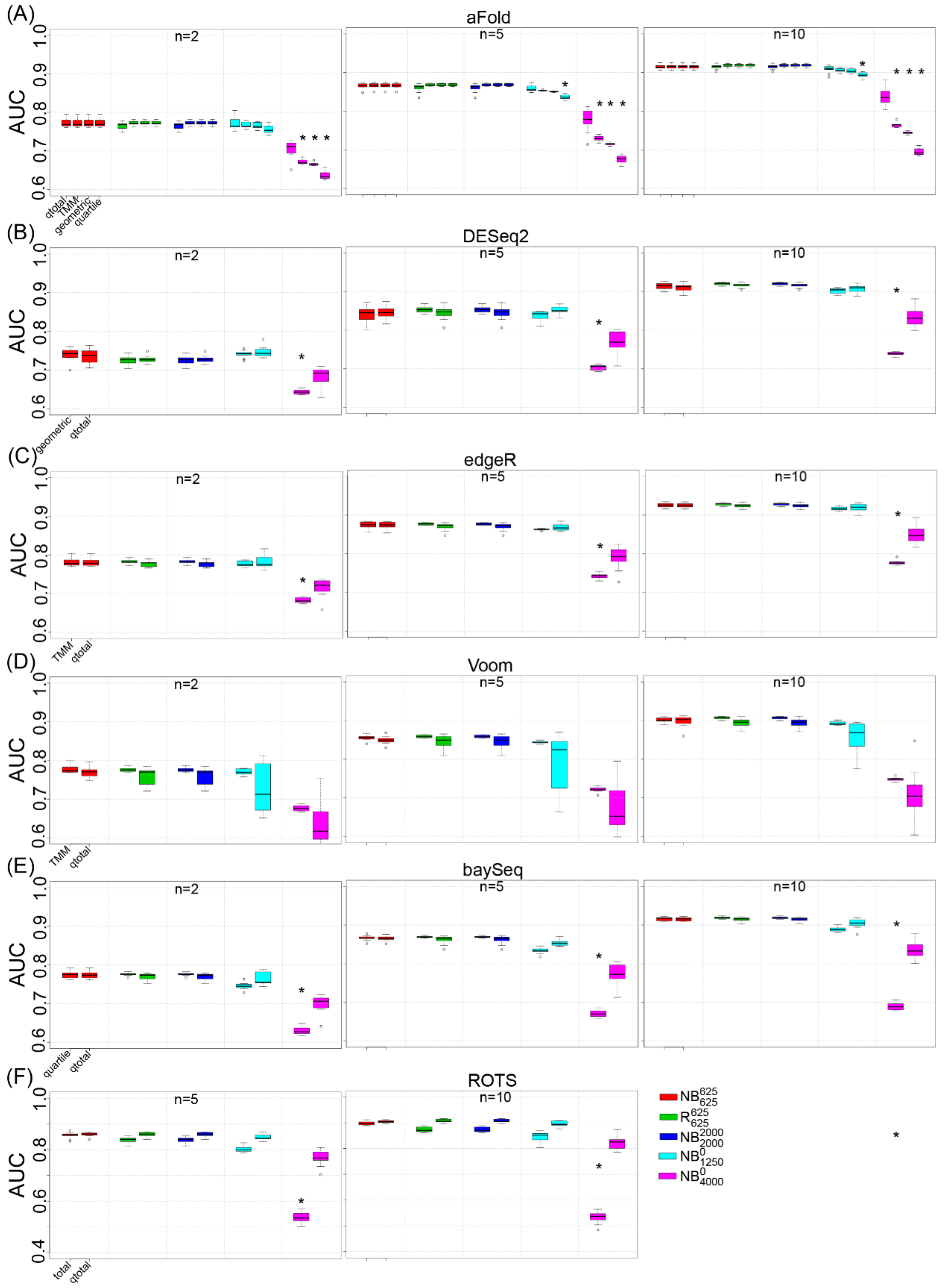

Figure S6. Additional AUC analysis on simulated data sets. AUC comparison on DE detection methods with different normalization approaches.

Asterisk indicates a statistically significant difference in AUC between qtotal and any of the other normalization methods.

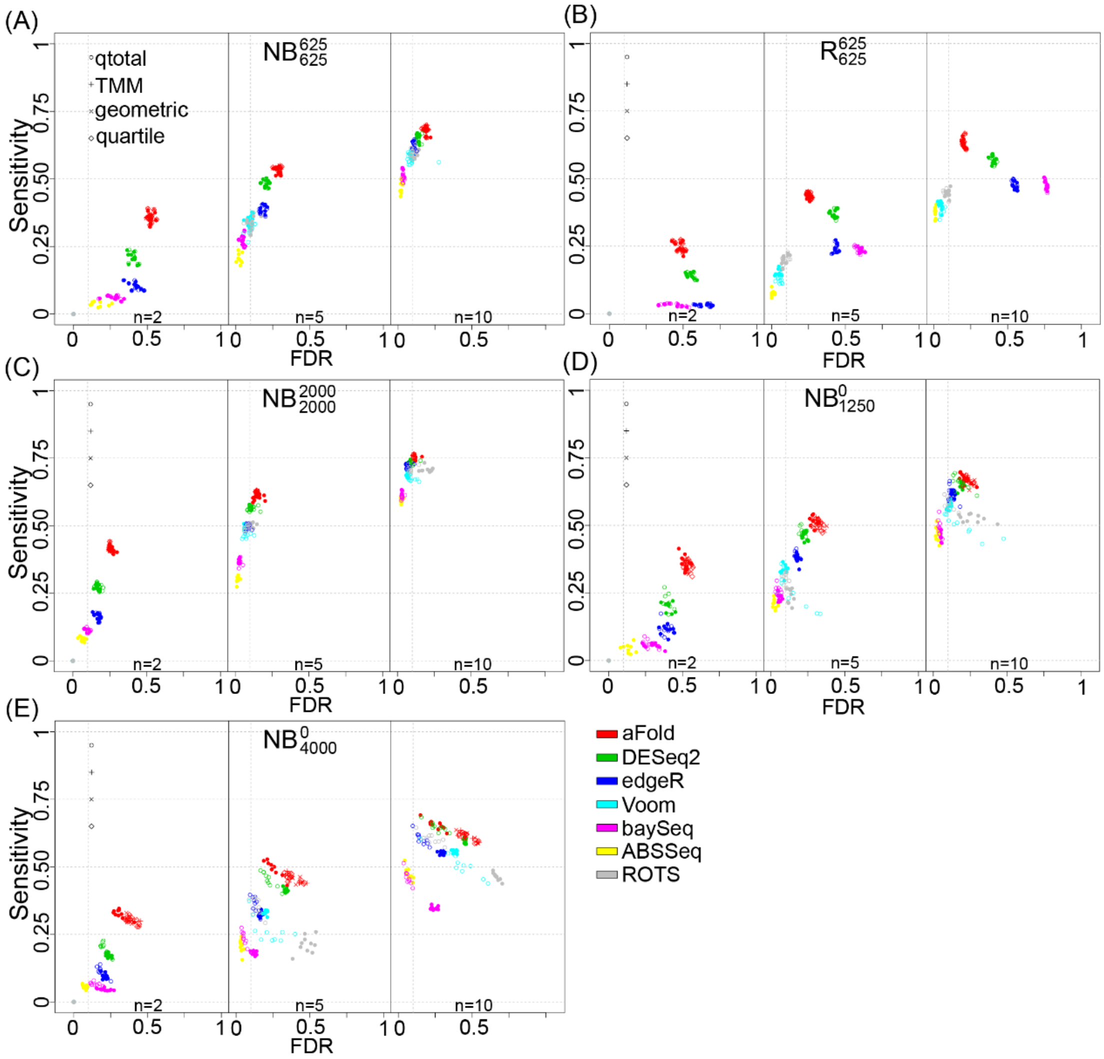

Figure S7. Additional Sensitivity and FDR analysis on simulated data sets.

Sensitivity and FDR are shown for DE detection methods with default and qtotal normalization approach. Results of aFold contain additional normalization approaches including TMM, geometric and quartile.

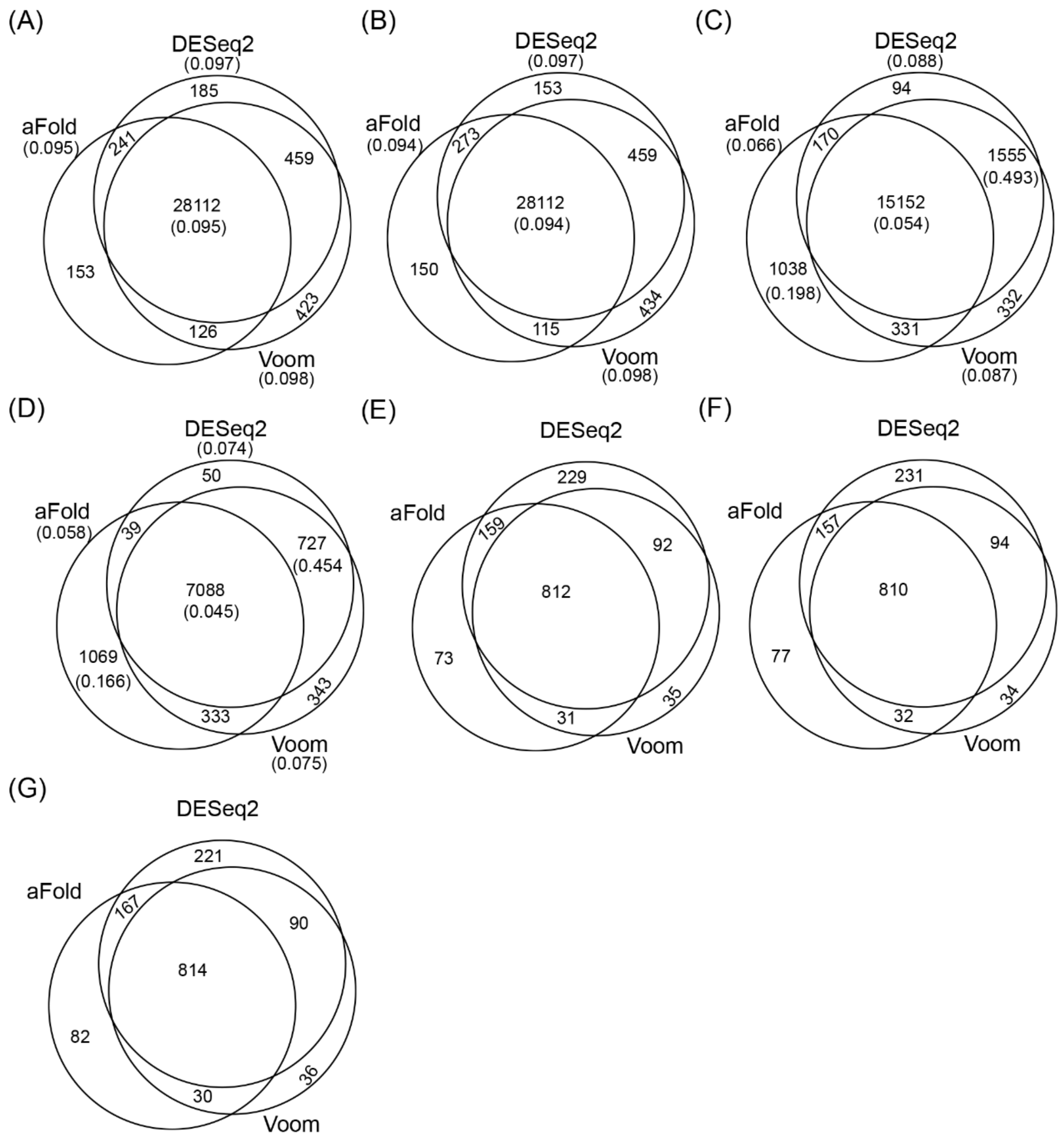

Figure S8. Venn diagram. Venn diagrams show number of DE identified by aFold, DESeq2 and Voom on four data sets: (A-B) the ABRF data set; (C) the SEQC data set; (D) the MAQC-II data set; (E-G) the Bottomly data set. aFold detects DE under three normalization procedures: qtotal (C-E), TMM (A and F) and geometric mean (B and G). Numbers in brackets indicate the eFDR.

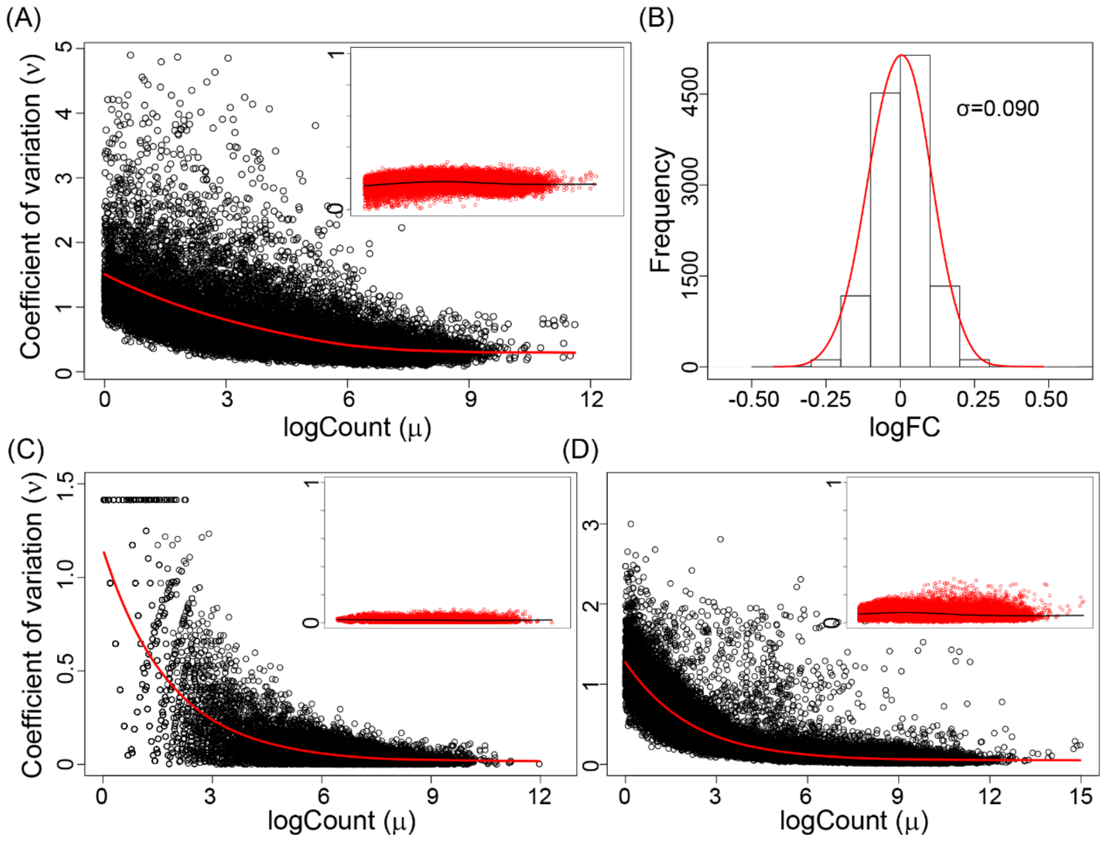

Figure S9. aFold modeling. Illustration based on the HapMap-CEU (A-B, large sample size n=24), Modencodefly (small sample size n=2, C) and ABRF (middle sample size n=9, D) data sets. (A,C-D) Mean-variance modeling and coefficient of variation (CV) normalization. Grey horizontal line indicates the baseline of CV. Red points in the inlet show CVs after uncertainty transformation. Red line (main panel) and black line (inlet) represent the fitted value of CV via *locfit*. (B) Distribution of aFold. Red line indicates a zero-centered normal distribution with an estimated standard deviation (SD) of 0.090.

References

1. Soneson C, Delorenzi M: **A comparison of methods for differential expression analysis of RNA-seq data.** *BMC Bioinformatics* 2013, **14:**91.
2. Pickrell JK, Marioni JC, Pai AA, Degner JF, Engelhardt BE, Nkadori E, Veyrieras J-B, Stephens M, Gilad Y, Pritchard JK: **Understanding mechanisms underlying human gene expression variation with RNA sequencing.** *Nature* 2010, **464:**768-772.
3. Shi L, Campbell G, Jones WD, Campagne F, Wen Z, Walker SJ, Su Z, Chu T-M, Goodsaid FM, Pusztai L: **The MicroArray Quality Control (MAQC)-II study of common practices for the development and validation of microarray-based predictive models.** *Nature biotechnology* 2010, **28:**827-838.
4. Shi L, Reid LH, Jones WD, Shippy R, Warrington JA, Baker SC, Collins PJ, De Longueville F, Kawasaki ES, Lee KY: **The MicroArray Quality Control (MAQC) project shows inter-and intraplatform reproducibility of gene expression measurements.** *Nature biotechnology* 2006, **24:**1151-1161.
5. Li S, Tighe SW, Nicolet CM, Grove D, Levy S, Farmerie W, Viale A, Wright C, Schweitzer PA, Gao Y: **Multi-platform assessment of transcriptome profiling using RNA-seq in the ABRF next-generation sequencing study.** *Nature biotechnology* 2014, **32:**915-925.
6. Bullard JH, Purdom E, Hansen KD, Dudoit S: **Evaluation of statistical methods for normalization and differential expression in mRNA-Seq experiments.** *BMC bioinformatics* 2010, **11:**94.
7. Frazee AC, Langmead B, Leek JT: **ReCount: a multi-experiment resource of analysis-ready RNA-seq gene count datasets.** *BMC Bioinformatics* 2011, **12:**449.
8. Dobin A, Davis CA, Schlesinger F, Drenkow J, Zaleski C, Jha S, Batut P, Chaisson M, Gingeras TR: **STAR: ultrafast universal RNA-seq aligner.** *Bioinformatics* 2013, **29:**15-21.
9. Celniker SE, Dillon LA, Gerstein MB, Gunsalus KC, Henikoff S, Karpen GH, Kellis M, Lai EC, Lieb JD, MacAlpine DM: **Unlocking the secrets of the genome.** *Nature* 2009, **459:**927-930.
10. Graveley BR, Brooks AN, Carlson JW, Duff MO, Landolin JM, Yang L, Artieri CG, van Baren MJ, Boley N, Booth BW: **The developmental transcriptome of Drosophila melanogaster.** *Nature* 2011, **471:**473-479.
11. Cheung VG, Nayak RR, Wang IX, Elwyn S, Cousins SM, Morley M, Spielman RS: **Polymorphic cis-and trans-regulation of human gene expression.** *PLoS biology* 2010, **8:**e1000480.
12. Law CW, Chen Y, Shi W, Smyth GK: **Voom: precision weights unlock linear model analysis tools for RNA-seq read counts.** *Genome biology* 2014, **15:**1.
13. Yang W, Rosenstiel PC, Schulenburg H: **ABSSeq: a new RNA-Seq analysis method based on modelling absolute expression differences.** *BMC Genomics* 2016, **17:**541.
14. Love MI, Huber W, Anders S: **Moderated estimation of fold change and dispersion for RNA-seq data with DESeq2.** *Genome biology* 2014, **15:**1.

15. Bottomly D, Walter N, Hunter JE, Darakjian P, Kawane S, Buck KJ, Searles RP, Mooney M, McWeeney SK, Hitzemann R: **Evaluating gene expression in C57BL/6J and DBA/2J mouse striatum using RNA-Seq and microarrays.** *PloS one* 2011, **6:**e17820.
16. Robinson MD, Oshlack A: **A scaling normalization method for differential expression analysis of RNA-seq data.** *Genome biology* 2010, **11:**1.
17. Oshlack A, Robinson MD, Young MD: **From RNA-seq reads to differential expression results.** *Genome biology* 2010, **11:**1.
18. BIPM, ISO: *Guide to the expression of uncertainty in measurement.* Geneve, Switzerland: International Organization for Standardization; 1995.
19. Loader C: **Local Regression and Likelihood. 1999.** *NY Springer-Verlag*.
20. Wu Z, Irizarry RA, Gentleman R, Martinez-Murillo F, Spencer F: **A model-based background adjustment for oligonucleotide expression arrays.** *Journal of the American statistical Association* 2004, **99:**909-917.
21. Feng J, Meyer CA, Wang Q, Liu JS, Liu XS, Zhang Y: **GFOLD: a generalized fold change for ranking differentially expressed genes from RNA-seq data.** *Bioinformatics* 2012, **28:**2782-2788.
22. Smyth GK: **Linear models and empirical bayes methods for assessing differential expression in microarray experiments.** *Stat Appl Genet Mol Biol* 2004, **3:**3.
23. Li H, Handsaker B, Wysoker A, Fennell T, Ruan J, Homer N, Marth G, Abecasis G, Durbin R: **The sequence alignment/map format and SAMtools.** *Bioinformatics* 2009, **25:**2078-2079.
